# Developing long-term conservation priority planning for medicinal plants in China by combining conservation status with diversity hotspot analyses and climate change prediction

**DOI:** 10.1186/s12915-022-01285-4

**Published:** 2022-04-21

**Authors:** Changying Xia, Yunfeng Huang, Yaodong Qi, Xudong Yang, Tiantian Xue, Renchuan Hu, Hongping Deng, Rainer W. Bussmann, Shengxiang Yu

**Affiliations:** 1 State Key Laboratory of Systematic and Evolutionary Botany, Institute of Botany, Chinese Academy of Sciences, Beijing, 100093 China; 2 University of Chinese Academy of Sciences, Beijing, 100049 China; 3 Chongqing Key Laboratory of Plant Resource Conservation and Germplasm Innovation, School of Life Sciences, Southwest University, Chongqing, 400715 China; 4 Guangxi Key Laboratory of Traditional Chinese Medicine Quality Standards, Nanning, 530022 China; 5 Guangxi Institute of Chinese Medicine and Pharmaceutical Science, Nanning, 530022 China; 6 Institute of Medicinal Plant Development, Chinese Academy of Medical Sciences and Peking Union Medical College, Beijing, 100193 China; 7 Department of Ethnobotany, Institute of Botany, Ilia State University, 0105 Tbilisi, Georgia

**Keywords:** Medicinal plants, Distribution pattern, Diversity hotspots, Climate change, Conservation effectiveness, Suitable habitat areas

## Abstract

**Background:**

Medicinal plants have always played an important role in the history of human health. However, the populations and sustainable use of medicinal plants have been severely affected by human activities and climate change. Little is known about the current conservation status and distribution pattern of medicinal plants. In this study, based on accurate geographical distribution information of 9756 medicinal plants, we identified diversity hotspots and conservation gaps, evaluated conservation effectiveness of nature reserves, and predicted suitable habitat areas for medicinal plants in China to provide scientific guidance for their long-term conservation and sustainable use.

**Results:**

A total of 150 diversity hotspot grid cells, mainly concentrated in central and southern China, were identified. These only accounted for 5% of the total distribution area but contained 96% of the medicinal plants of the country. The hotspot grid cells included all traditional hotspot areas, but we also detected three new hotspots, namely Mufu-Lushan Mountains, Tianshan-Altai Mountains, and Changbai Mountains. The current national and provincial nature reserves protect 125 hotspot grid cells, which harbor 94% of all medicinal plants. However, 25 hotspot grid cells, distributed in the Tianshan-Altai Mountains and Hengduan Mountains, are located outside the national and provincial nature reserves. An analysis of the predicted effects of climate change indicated that the suitable habitat areas will shift from southern to northern China, and that southern China will face a considerable loss of suitable habitat areas, while the east and west parts of China will encompass remarkably more suitable habitat areas in the future.

**Conclusions:**

The current conservation networks have achieved high conservation effectiveness with regard to medicinal plants; however, the conservation gaps we identified should not be neglected, and conservation planning needs to take into account the predicted shifts of some hotspots of medicinal plants due to climate change.

**Supplementary Information:**

The online version contains supplementary material available at 10.1186/s12915-022-01285-4.

## Background

Medicinal plants have long been used to treat or prevent diseases and are already well documented in ancient Egypt, India, and China [1–4]. The World Health Organization (WHO) reported that herbal medicine still meets the health needs of about 80% of the global population, and especially millions of people in rural areas of developing countries [1]. So far, about 35,000 plant species have been documented as used as medicinal plants [1]. Unfortunately, due to overexploitation, expansion of alien invasive species and climate change, medicinal plants are facing an increasing risk of habitat destruction and extinction [5–7].

Hotspot identification and gap analysis are useful methods to evaluate conservation effectiveness and select conservation priority areas [8–12]. Biodiversity hotspot determination is highly commended to consider multiple conservation indicators, for example, all species, endemic species, threatened species, and national protected species [13, 14]. However, there are many studies on biodiversity hotspots that are based only on limited conservation indicators and using single species richness algorithm [15–17], which may overlook other aspects of biodiversity attributes [2, 8, 13]. Currently, the top 5% richness algorithm and complementary algorithm are the two algorithms most commonly used to identify hotspots [2, 8, 18]. The former identifies hotspots mainly based on species richness [8], while the latter pays more attention to species complementarity [18]. Taking both species richness and species complementarity into consideration is expected to provide more comprehensive distribution patterns for identifying priority areas of biodiversity conservation than the use of a single diversity research method [19].

In recent years, many studies have focused on distribution patterns of biodiversity hotspots and gaps for the identification of conservation priority areas [8, 11, 20, 21]. Zhang et al. identified conservation gaps for threatened plants by overlapping the distribution of species richness with the existing conservation network in China composed of national nature reserves and provincial nature reserves and found that only 27.5% of the species distribution areas were covered [21]. Huang et al. identified hotspots and conservation gaps for Chinese endemic seed plant species based on their distribution patterns and found that only 26.48% of hotspot areas were covered by nature reserves [11]. However, still little is known about the distribution patterns and conservation status of medicinal plants in China [2, 22–25]. For example, Li et al. analyzed the distribution patterns of 3150 endemic medicinal plants in China, based on provincial distribution data [22]. Chi et al. studied the distribution pattern of 535 threatened medicinal plants in China and identified hotspots and conservation gaps based on county-level distribution data [2]. Zhang et al. and Chi et al. evaluated the conservation effectiveness of national nature reserves in northern China and central China on medicinal vascular plants, respectively [24, 25]. Overall, due to their localized region, small number of plant species, and low-resolution occurrence data, these previous studies failed to present the distribution patterns, conservation effectiveness, and gaps of Chinese medicinal plants as a whole, let alone provided a baseline for conservation priority planning.

Climate change has been considered as a major threat to biodiversity in the twenty-first century because it may cause the loss of biodiversity, termination of evolutionary potential, and disruption of ecological services [26–28]. Previous studies have indicated that the changing climate of the past century has already resulted in a globally consistent fingerprint of poleward and/or upward shifts in species distributions [29–32]. Therefore, it is necessary to consider the impact of climate change on medicinal plants for the protection planning of medicinal plants. The MaxEnt species distribution model has been widely applied to predict species ranges and vegetation shifts under climate change, which in turn can provide new information for biodiversity conservation [28, 33–35]. Many studies have been trying to predict the distribution patterns of current and future suitable habitat areas for medicinal plants using MaxEnt in the context of climate change. This showed that many medicinal plants are expected to be adversely affected by the expected climate change, and their suitable range would be shrinking or moving substantially [33, 36–38]. Nevertheless, MaxEnt has only been used to predict the suitable habitat areas of few Chinese medicinal plant species under climate change scenarios [29, 39–41], which may not reflect the impact of climate change on the diversity of all the medicinal plants in China.

**Fig. 1:**
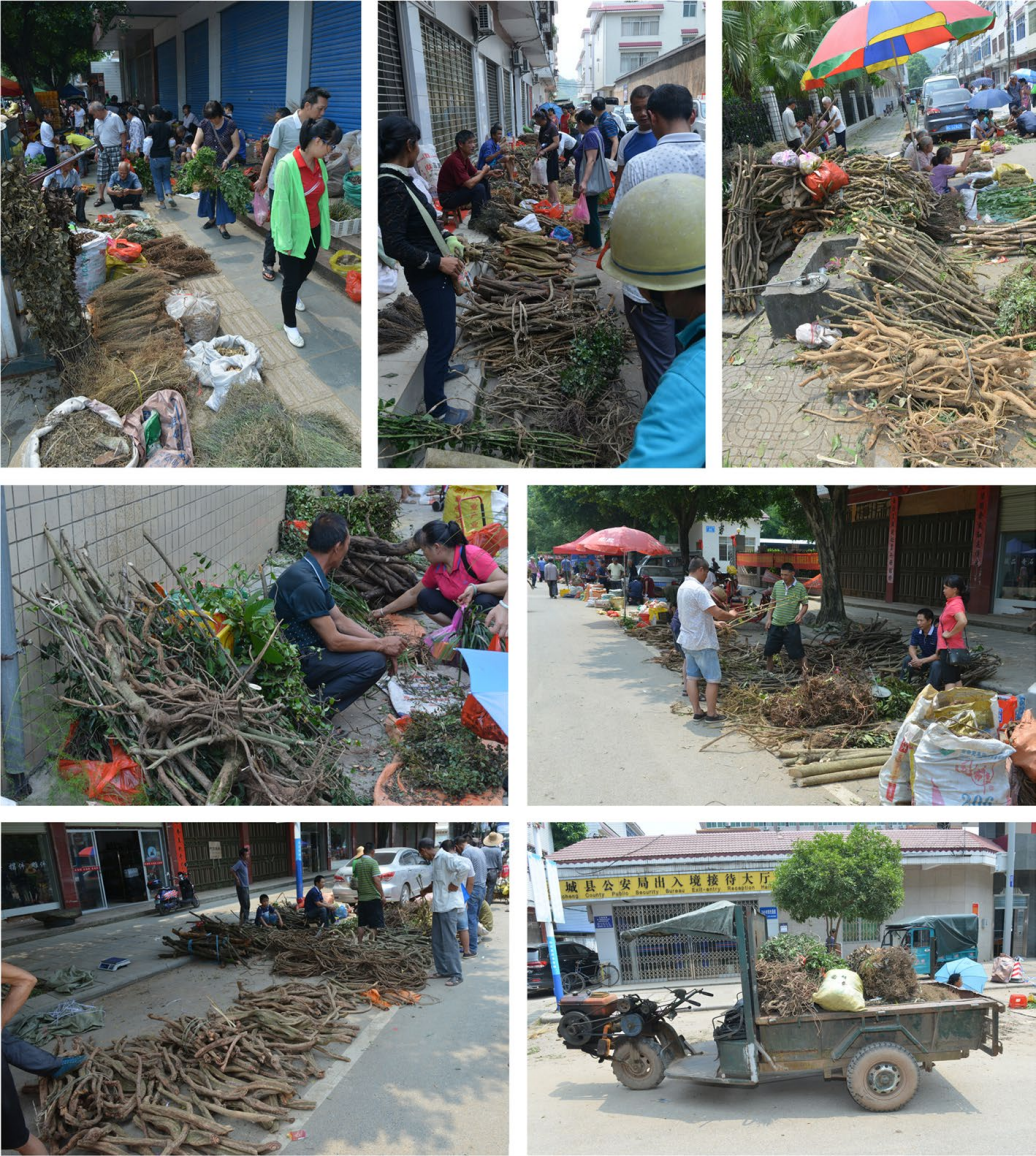
Dragon Boat Festival wild medicinal plants market in Gongcheng, Guangxi, China, showing that wide medicinal plants are confronting with over collection and consumption by local people

China, as one of the richest countries in plant resources, harbors a total of 35,784 higher plant species [42], including 9756 species of medicinal plants, belonging to 285 families and 2087 genera. It is estimated that about 70% of the commonly used herbal medicines in China are still wild harvested [7]. Long-term overexploitation, habitat degradation and loss, and climate change have severely damaged the wild resources of medicinal plants and exacerbated the risk of extinction to many medicinal plants [7, 43] (Fig. 1, Additional file 1: Fig. S1). Extinction in the wild or local, or significant reduction of the number of medicinal plants will directly lead to the loss of this precious genetic resource. Therefore, the protection of wild medicinal plant resources has become increasingly urgent.

Highly representative and accurate conservation priority areas can be identified using comprehensive methods based on multiple diversity indicators and high-resolution geographic distribution data [2, 11, 44, 45]. In this study, for the first time, we determined a large-scale high-resolution distribution pattern of medicinal plants in China using comprehensive methods based on the latest list of medicinal plant species, a large set of precise occurrence data, and multiple diversity indicators. We aimed to (1) identify the distribution patterns and diversity hotspots of medicinal plants; (2) evaluate the conservation effectiveness and identify conservation gaps in the existing nature reserves; (3) predict the current and future distribution of suitable habitat areas for medicinal plants and find stable suitable habitat areas for long-term conservation; and (4) put forward suggestions for biodiversity conservation of medicinal plants.

## Results

### Distribution patterns of different algorithms and groups

**Fig. 2:**
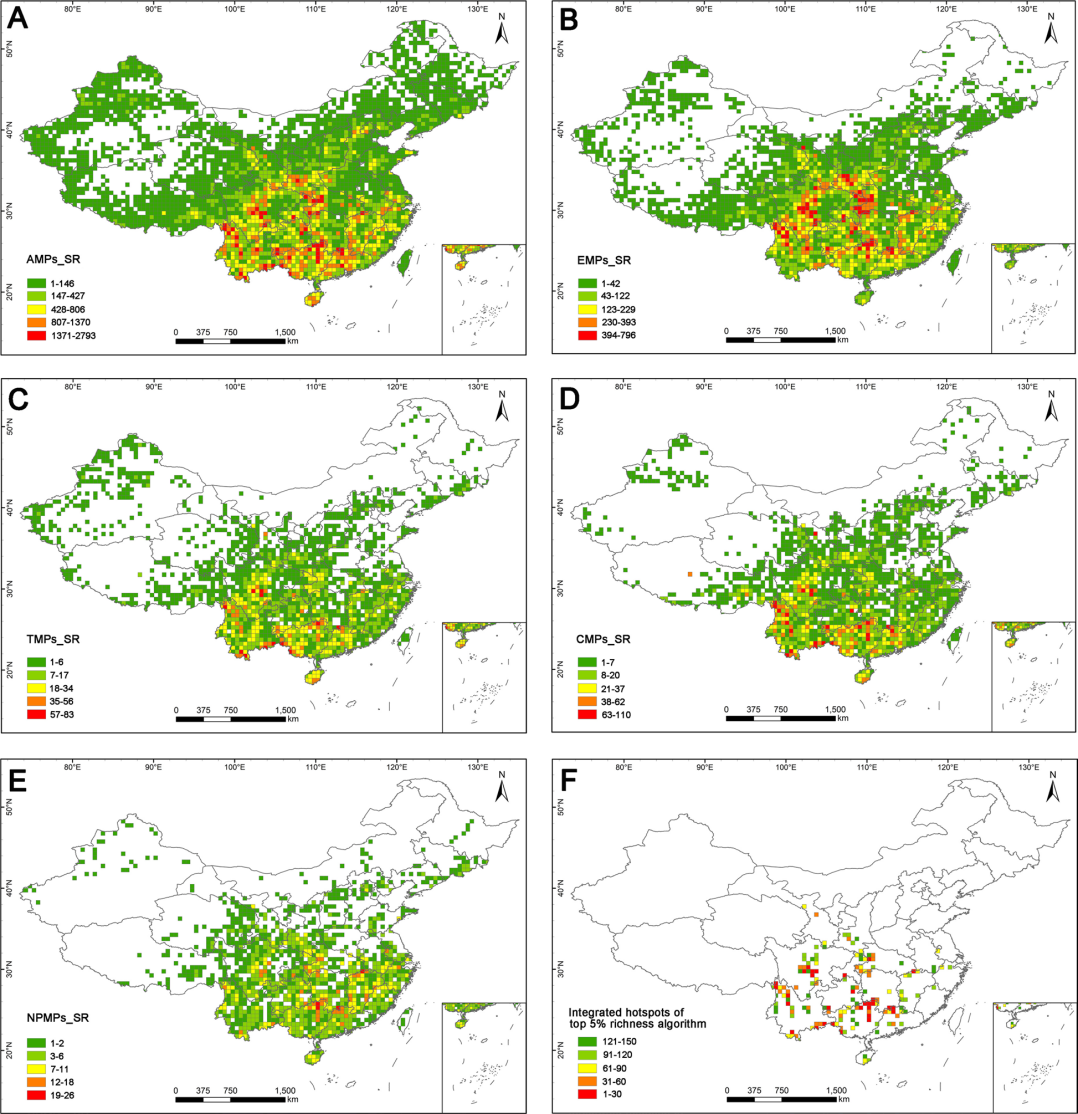
Distribution patterns of species richness of medicinal plants for: **A** Species richness of all medicinal plants (AMPs_SR). **B** Species richness of endemic medicinal plants (EMPs_SR). **C** Species richness of threatened medicinal plants (TMPs_SR). **D** Species richness of CITES listed medicinal plants (CMPs_SR). **E** Species richness of national protected medicinal plants (NPMPs_SR). **F** Species richness of integrated hotspots of the top 5% richness algorithm

All geographical names listed in the results could refer to Additional file 1: Figs. S2, S3. According to the top 5% richness algorithm, medicinal plants were mainly distributed in south-western and southern China (Fig. 2A–E, Additional file 1: Figs. S4-S8). All medicinal plants and endemic medicinal plants showed a concentrated distribution in the surrounding areas of Sichuan Basin (Fig. 2A, B, Additional file 1: Figs. S4, S5). For all medicinal plants, diversity was also concentrated in the Hengduan Mountains (north-western Yunnan), the junction area between Guizhou and Guangxi, the boundary areas between Vietnam and China, and Nanling Mountains (Fig. 2A, Additional file 1: Fig. S4). The endemic medicinal plants were also mainly located in the Hengduan Mountains (north-western Yunnan) and the Nanling Mountains (Fig. 2B, Additional file 1: Fig. S5). The threatened medicinal plants and CITES (Convention on International Trade in Endangered Species of Wild Fauna and Flora) listed medicinal plants shared a similar distribution pattern and were mainly confined to the Hengduan Mountains, Nanling Mountains, and the boundary areas between Vietnam and China (Fig. 2C, D, Additional file 1: Figs. S6, S7). The distribution patterns of national protected medicinal plants were different from the above groups and were mainly located in south-eastern China, especially in the Nanling Mountains (Fig. 2E, Additional file 1: Fig. S8). Correlation analysis indicated that there were very strong correlations (*r* = 0.81–0.94, *p* < 0.01) between distribution patterns of all five groups according to the top 5% richness algorithm, except for a strong correlation (*r* = 0.74, *p* < 0.01) between CITES listed medicinal plants and national protected medicinal plants (Fig. 4).

**Fig. 3:**
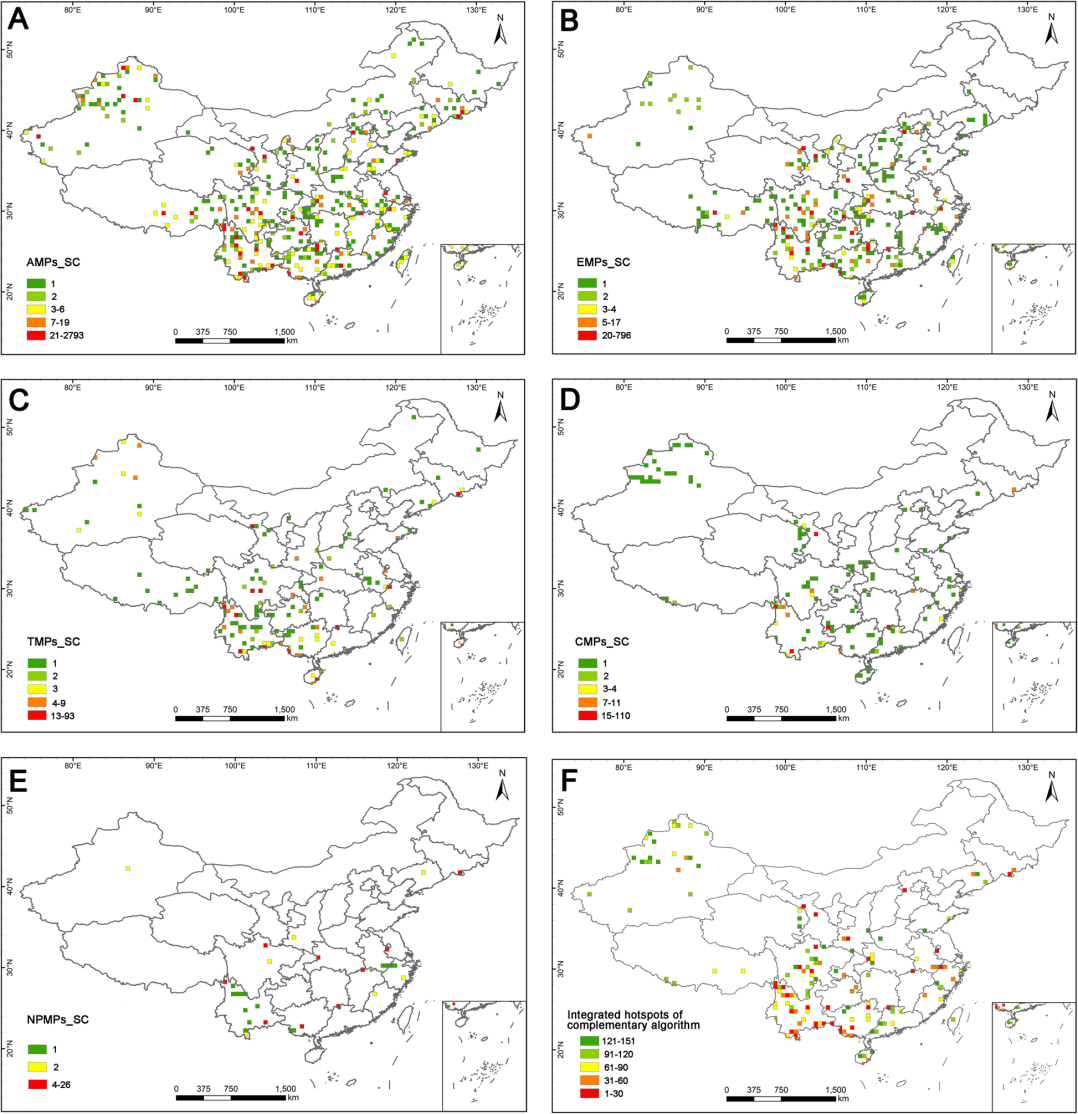
Distribution patterns of species complementarity of medicinal plants for: **A** Species richness of all medicinal plants (AMPs_SR). **B** Species richness of endemic medicinal plants (EMPs_SR). **C** Species richness of threatened medicinal plants (TMPs_SR). **D** Species richness of CITES listed medicinal plants (CMPs_SR). **E** Species richness of national protected medicinal plants (NPMPs_SR). **F** Integrated hotspots of the complementary algorithm

**Fig. 4:**
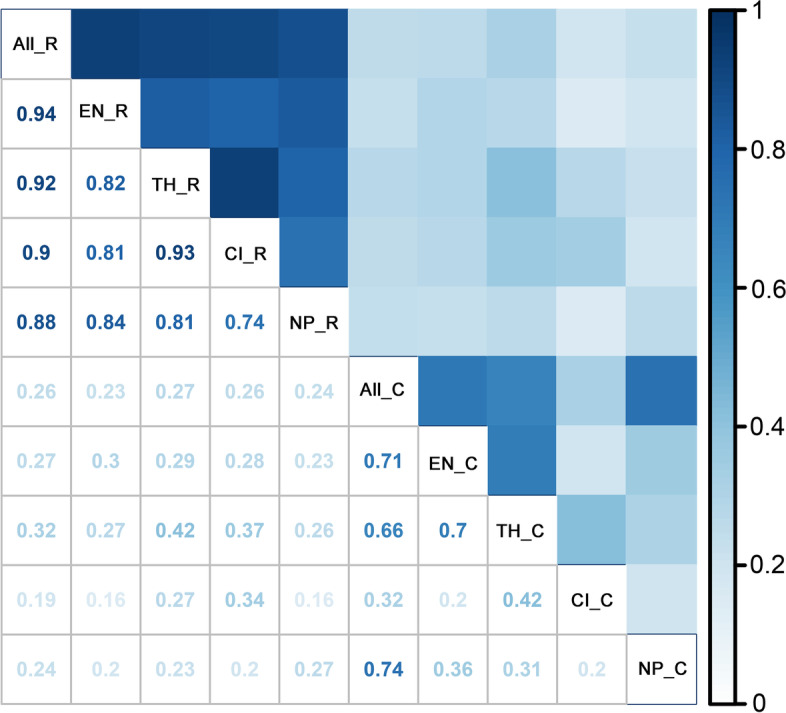
The correlogram of the distribution pattern of the top 5% richness algorithm (R) and complementary algorithm (C) of all medicinal plants (All), endemic medicinal plants (EN), threatened medicinal plants (TH), CITES listed medicinal plants (CI), and national protected medicinal plants (NP)

The distribution patterns of the complementary algorithm appeared more dispersed however (Fig. 3A–E). The grid cells with high species complementarity were scattered throughout the country, and especially concentrated in south-western and southern China (Fig. 3A–E). There were strong correlations (0.66–0.74, *P* < 0.01, Fig. 4) between distribution patterns of all medicinal plants and endemic medicinal plants, or threatened medicinal plants, or national protected medicinal plants, endemic medicinal plants, and threatened medicinal plants in the complementary algorithm. No significant correlation was identified between other groups (0.2–0.42, *P* < 0.01, Fig. 4). In general, the grid cells with high species richness and species complementarity were all mainly located in south-eastern and southern China. However, correlation analysis of the two algorithms indicated weak or very weak correlations (0.16–0.37, *P* < 0.01, Fig. 4) between distribution patterns of the different groups, except a moderate correlation (0.42, *P* < 0.01) between the threatened ones (Fig. 4).

### Distribution patterns of hotspots of medicinal plants

The integrated hotspots identified by the top 5% richness algorithm were mainly distributed in the surrounding areas of Sichuan Basin (especially in its west and east), Hengduan Mountains (north-western Yunnan), the junction area between Guizhou and Guangxi, the Xishuangbanna region, the boundary areas between Vietnam and China, Nanling Mountains, Hainan Island, Luoxiao Mountains, Mufu-Lushan Mountains, and Tianmu Mountains (Fig. 2F). In these areas, there were a total of 150 hotspot grid cells, which harbored 8779 medicinal plant species (90%), including 3024 endemic medicinal plant species (94%), 528 threatened medicinal plant species (85%), 330 CITES listed medicinal plant species (96%), and 68 national protected medicinal plant species (89%, Table 1).

**Table 1:** Statistics of China, hotspots, grid cells covered by nature reserves (NRs) focusing on the whole country for all medicinal plants (AMPs), endemic medicinal plants (EMPs), threatened medicinal plants (TMPs), CITES listed medicinal plants (CMPs) and national protected medicinal plants (NPMPs). (Note: The denominators of all the proportions are the total number of corresponding groups)

	Statistics for China and hotspots				Statistics for diversity hotspots						Statistics for grid cells covered by NRs focusing on the whole country		
Taxa	China	Top 5% richness algorithm hotspots	Complementary algorithm hotspots	Diversity hotspots	Hotspots covered by NNRs	Hotspots covered by PNRs	Hotspots covered by NNRs and PNRs	Conservation gaps of NNRs	Conservation gaps of PNRs	Conservation gaps of NNRs and PNRs	All grid cells covered by NNRs	All grid cells covered by PNRs	All grid cells covered by NNRs and PNRs
AMPs	9756	8779/90%	9405/96%	9338/96%	8890/91%	8714/89%	9135/94%	7965/82%	8679/89%	6652/68%	9431/97%	9436/97%	9641/99%
EMPs	3205	3024/94%	3089/96%	3084/96%	2973/93%	2902/91%	3045/95%	2490/78%	2784/97%	1898/59%	3109/97%	3120/97%	3177/99%
TMPs	620	528/85%	596/96%	580/94%	536/86%	508/82%	560/90%	423/68%	503/81%	321/52%	589/95%	570/92%	606/98%
CMPs	344	330/96%	344/100%	339/99%	327/95%	314/91%	333/97%	295/86%	324/94%	264/77%	334/97%	332/97%	343/100%
NPMPs	76	68/89%	76/100%	76/100%	72/95%	68/89%	73/96%	61/80%	70/92%	48/63%	72/95%	73/96%	74/97%
The number of grid cells	3047	150/5%	151/5%	150/5%	101/3%	86/3%	125/4%	49/2%	64/2%	25/1%	1042/34%	1116/37%	1755/58%

*NNRs* national nature reserves and *PNRs* provincial nature reserves

However, the integrated hotspots identified by the complementary algorithm were distributed more discretely (Fig. 3F). Except for some hotspot areas shared with the top 5% richness algorithm (e.g., Hengduan Mountains, Bashan-Wushan Mountains, Nanling Mountains and the boundary areas between Vietnam and China), the complementary algorithm also identified some new hotspot areas, such as the Tianshan-Altai Mountains, Changbai Mountains, eastern Xizang, eastern Zhejiang, and Taihang Mountains (Fig. 3F). These integrated hotspots harbored a total of 151 hotspot grid cells, which contained 9405 medicinal plant species (96%), 3089 endemic medicinal plant species (96%), 596 threatened medicinal plant species (96%), 344 CITES listed medicinal plant species (100%), and 76 national protected medicinal plant species (100%, Table 1).

**Fig. 5:**
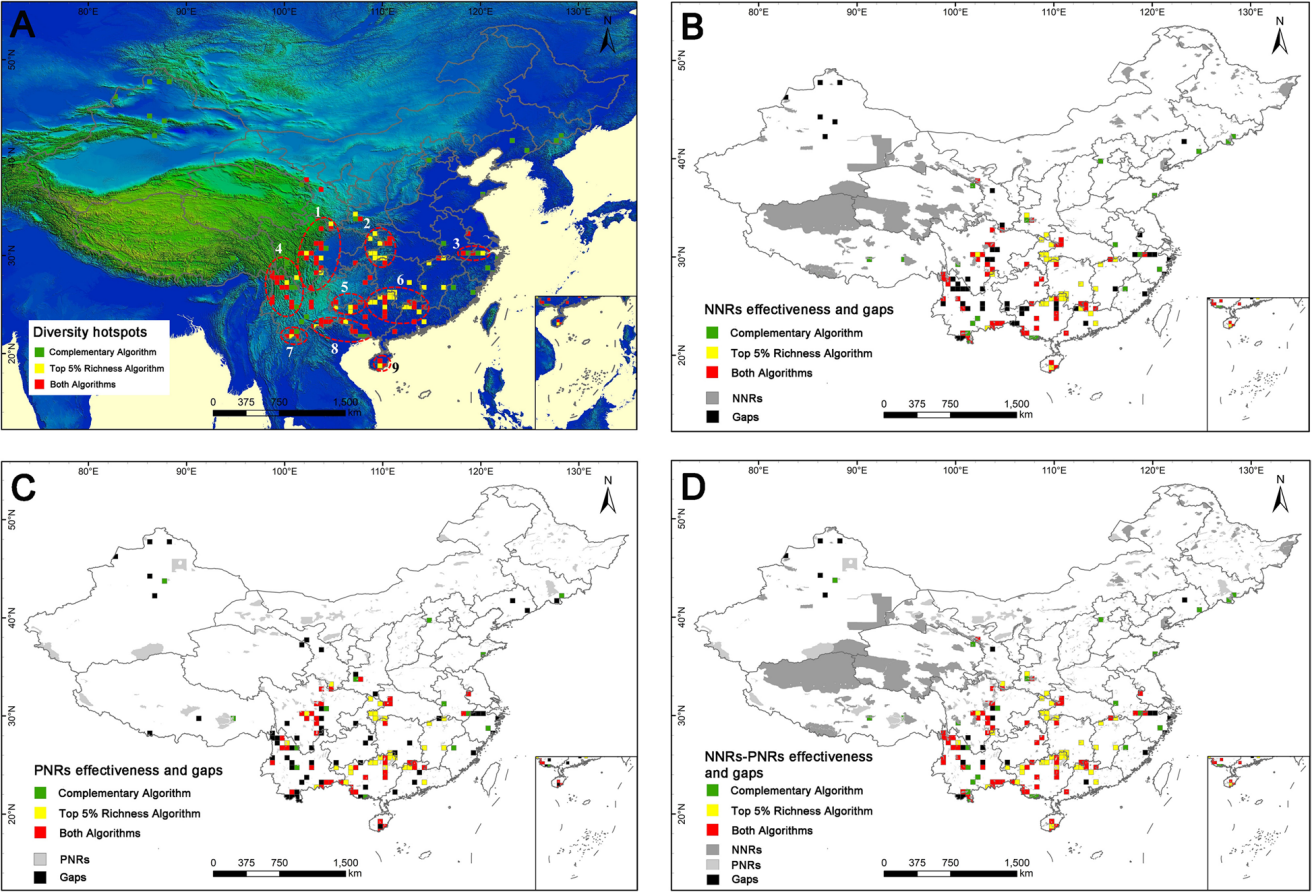
Distribution patterns of final diversity hotspots, conservation effectiveness, and gaps. **A** Comprehensive hotspot grid cells obtained through the synthesis of top 5% richness algorithm and complementary algorithm. The red elliptic circles refer to the 9 diversity hotspot areas: 1. Hengduan Mountains (western Sichuan), 2. Bashan-Wushan Mountains, 3. Tianmu Mountain, 4. Hengduan Mountains (north-western Yunnan), 5. the junction area between Guizhou and Guangxi, 6. Nanling Mountains, 7. the Xishuangbanna region, 8. the boundary areas between Vietnam and China, 9. Hainan Island. **B** Conservation effectiveness and gaps of national nature reserves (NNRs), with conservation gaps of national nature reserves in black. **C** Conservation effectiveness and gaps of provincial nature reserves (PNRs), with conservation gaps of provincial nature reserves in black. **D** Conservation effectiveness and gaps of national nature reserves (NNRs) and provincial nature reserves (PNRs), with conservation gaps of national and provincial nature reserves in black

Finally, based on both algorithms, final diversity hotspots were confirmed, including a total of 150 hotspot grid cells with higher species richness and species complementarity (Fig. 5A). These hotspot grid cells only accounted for 5% of the total grid cells with occurrence data, but harbored 9338 medicinal plant species (96%), 3084 endemic medicinal plant species (96%), 580 threatened medicinal plant species (94%), 339 CITES listed medicinal plant species (99%), and 76 national protected medicinal plant species (100%, Table 1). In general, most of these hotspot grid cells were distributed in central, southern, and south-eastern China, and only a few hotspot grid cells were scattered in north-western and north-eastern China. Based on the distribution pattern of mountains, rivers, and administrative divisions [46] (Additional file 1: Figs. S2, S3), 150 hotspot grid cells were mainly distributed in 9 key hotspot areas (Fig. 5A, Additional file 2: Table S2.1), including (1) Hengduan Mountains (western Sichuan), (2) Bashan-Wushan Mountains, (3) Tianmu Mountain, (4) Hengduan Mountains (north-western Yunnan), (5) the junction area between Guizhou and Guangxi, (6) Nanling Mountains, (7) the Xishuangbanna region, (8) the boundary areas between Vietnam and China, and (9) Hainan Island.

### Conservation effectiveness and gaps of current conservation networks

Conservation effectiveness analysis indicated that 101 out of 150 hotspot grid cells were protected by national nature reserves (Fig. 5B), which harbored 8890 medicinal plant species (91%, Table 1). Most protected hotspot grid cells were concentrated in the Hengduan Mountains, Bashan-Wushan Mountains, the junction area between Guizhou and Guangxi, Nanling Mountains, the Xishuangbanna region, the boundary areas between Vietnam and China, and Hainan Island (Fig. 5B). Eighty-six hotspot grid cells were covered by provincial nature reserves, harboring 8714 medicinal plant species (89%), and were mostly distributed in the Hengduan Mountains, Bashan-Wushan Mountains, the junction area between Guizhou and Guangxi, Nanling Mountains, the boundary areas between Vietnam and China, and Hainan Island (Fig. 5C, Table 1). In total 125 hotspot grid cells were covered by national and provincial nature reserves, mostly concentrated in the Hengduan Mountains, Bashan-Wushan Mountains, the junction area between Guizhou and Guangxi, Nanling Mountains, and the boundary areas between Vietnam and China (Fig. 5D). These hotspot grid cells harbored 9135 medicinal plant species (94%), 3045 endemic medicinal plant species (95%), 560 threatened medicinal plant species (90%), 333 CITES listed medicinal plant species (97%), and 73 national protected medicinal plant species (96%, Table 1).

There were 49 hotspot grid cells identified as conservation gaps of national nature reserves and mainly distributed in the Tianshan-Altai Mountains, Hengduan Mountains, the junction area between Guizhou and Guangxi, Nanling Mountains, and Tianmu Mountains (Fig. 5B), of which contained 7965 medicinal plant species (82%, Table 1). In addition, the gap analysis also showed that provincial nature reserves covered some conservation gaps of national nature reserves in key hotspot areas, such as Hengduan Mountains, the junction area between Guangzhou and Guangxi, and Nanling Mountains (Fig. 5C). Nevertheless, 25 hotspot grid cells mainly distributed in the Tianshan-Altai Mountains, Hengduan Mountains, the Xishuangbanna region, and Tianmu Mountains still remained outside the nature reserves (Fig. 5D), which recorded 6652 medicinal plant species (68%), 1898 endemic medicinal plant species (59%), 321 threatened medicinal plant species (52%), 264 CITES listed medicinal plant species (77%), and 48 national protected medicinal plant species (63%) (Fig. 5D, Table 1).

**Fig. 6:**
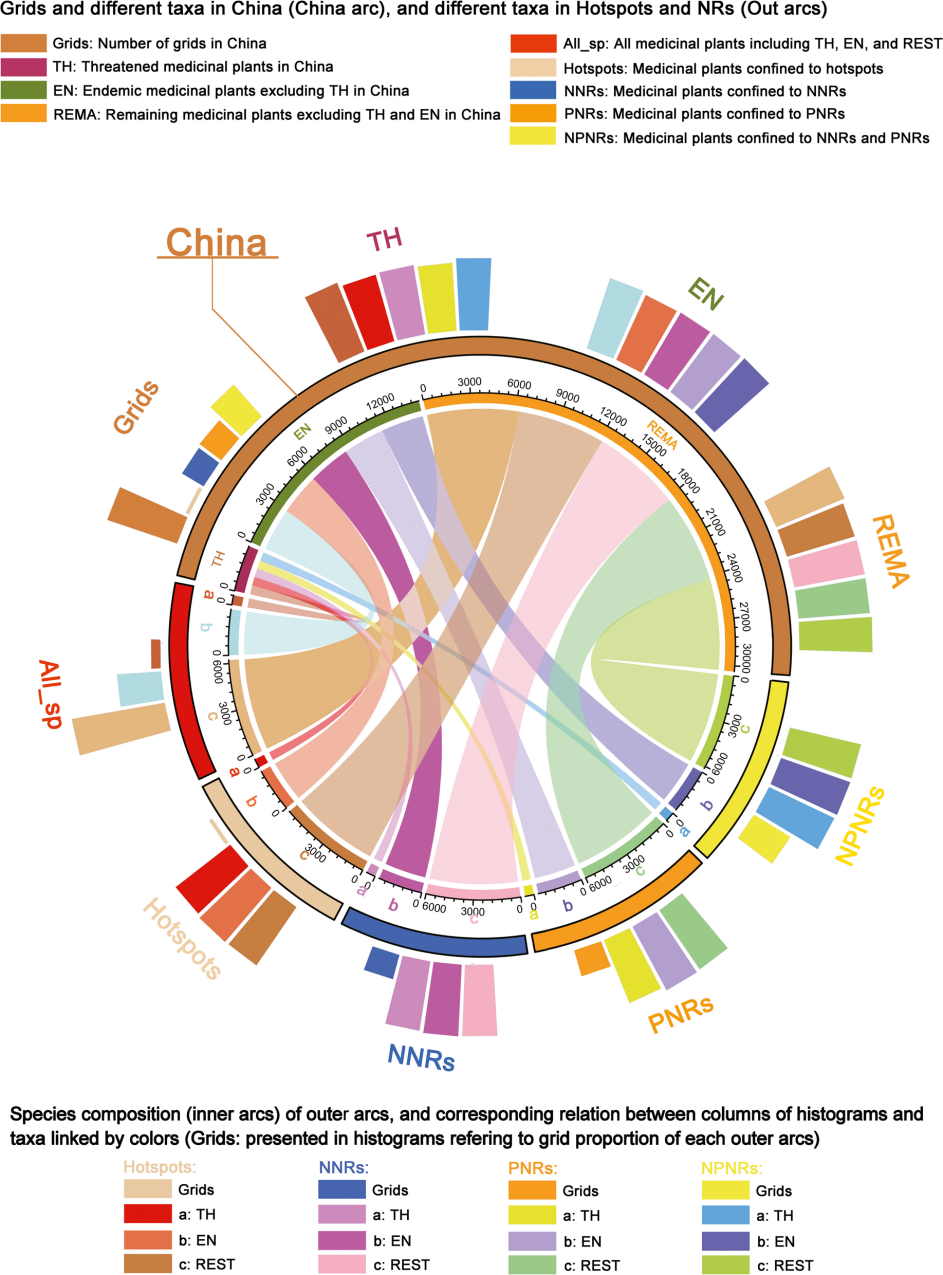
Chord diagram and circular barplot showing the number of grid cells, species composition of different areas (hotspots, national nature reserves (NNRs), and provincial nature reserves (PNRs)). The inner arcs are linked up with circular barplot by the same color to represent the same group. The colored segments in the inner arc represented the number of medicinal plants of certain group in different areas. The part of all species (All_sp) presented species composition of all medicinal plants in inner segments and presented proportion of different group in outer histogram

**Fig. 7:**
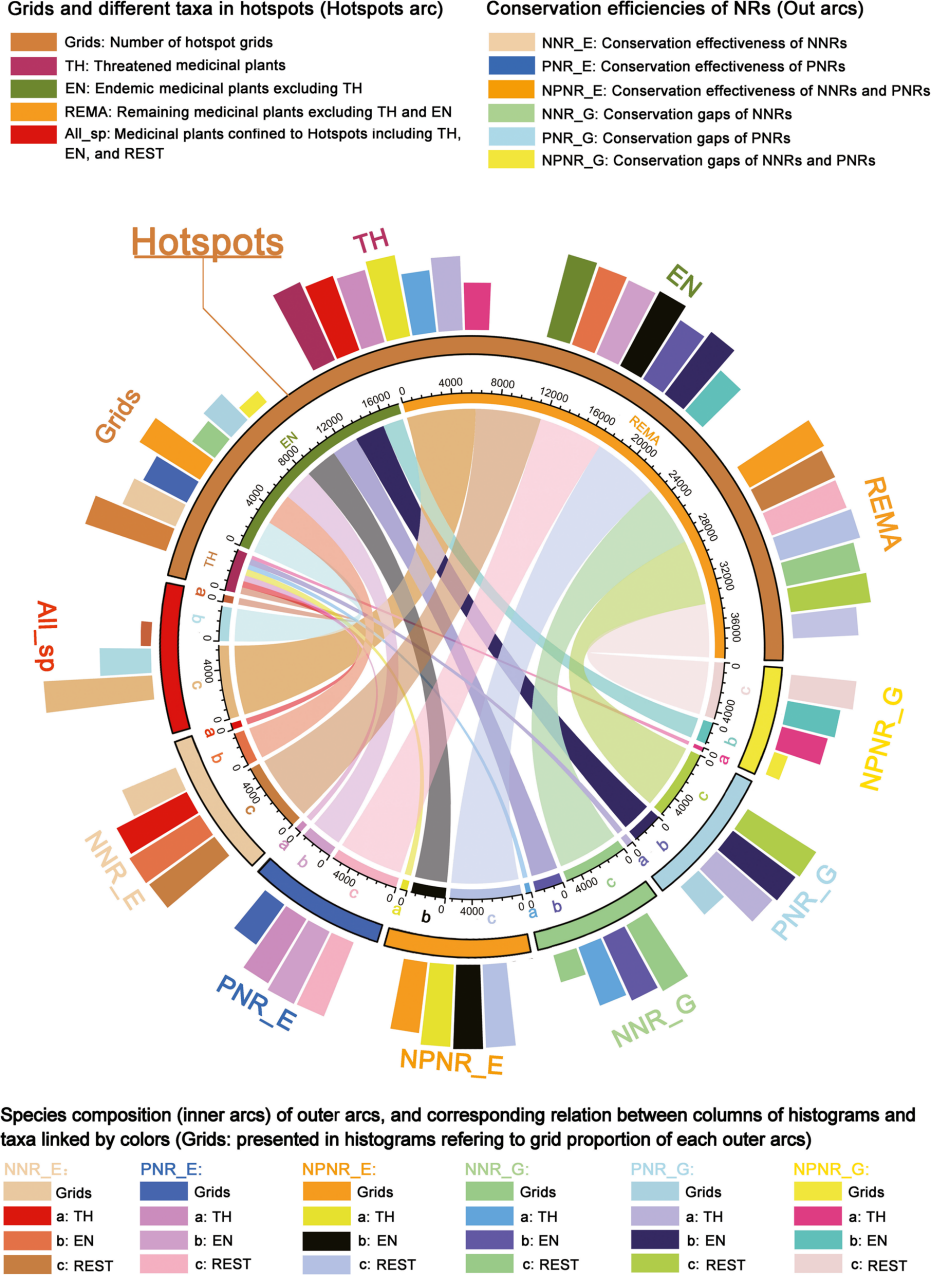
Chord diagram and circular barplot showing the number of grid cells and species composition of the diversity hotspots, conservation effectiveness (for hotspots), and conservation gaps (for hotspots) of national nature reserves (NNRs) and provincial nature reserves (PNRs). The inner arcs and circular barplot are linked in the same color to represent the same group. The colored segments in the inner arc represented the number of medicinal plants of certain group in different areas. The ratio of the number of medicinal plants of each group in hotspots to the number of all medicinal plants in hotspots is shown in All_sp

As for species composition in hotspot grid cells and nature reserves, the hotspot grid cells covered only 5% of the total area (total grid cells with occurrence data) but harbored 96% of all medicinal plant species (Fig. 6, Table 1). However, national nature reserves covered 34% of the total area and contained 97% of all medicinal plant species; provincial nature reserves covered 37% of the total area and contained 97% of all medicinal plant species (Fig. 6, Table 1). More details on species composition and land areas of conservation effectiveness and conservation gaps are presented in Figs. 6 and 7 and Additional file 2: Table S2.2.

### Suitable habitat areas of threatened medicinal plants

**Fig. 8:**
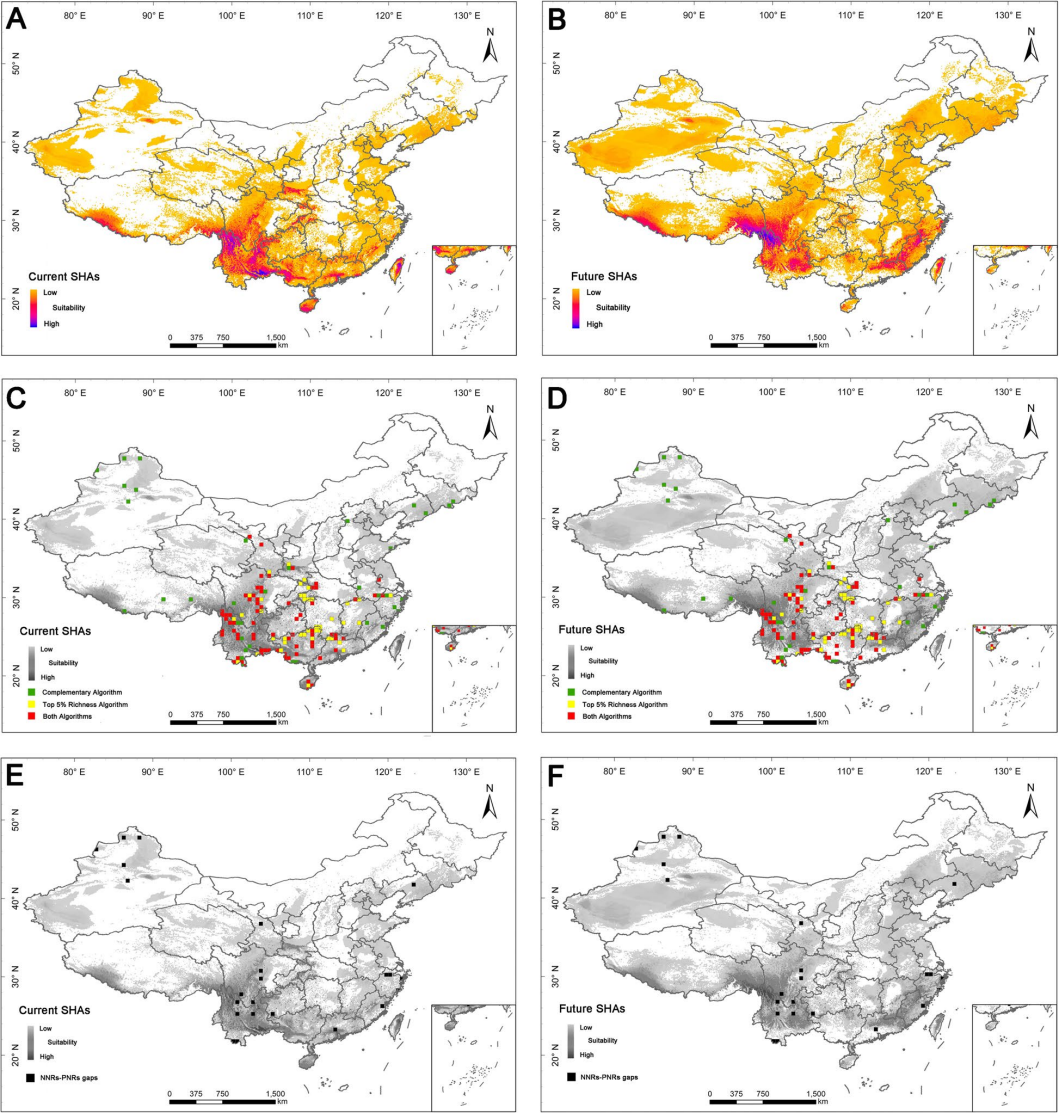
Distribution patterns of potential suitable habitat areas, and superposition of suitable habitat areas with hotspot grid cells and conservation gaps. **A** Potential suitable habitat areas (SHAs) under the current climate scenario. **B** Potential suitable habitat areas (SHAs) under the future climate scenario in 2100s. **C** Superposition of current suitable habitat areas (SHAs) with hotspot grid cells. **D** Superposition of future suitable habitat areas (SHAs) with hotspot grid cells. **E** Superposition of current suitable habitat areas (SHAs) with national and provincial conservation gaps. **F** Superposition of future suitable habitat areas (SHAs) with national and provincial conservation gaps

The prediction results showed that all models used in the analysis produced high values (0.907–1.000, Additional file 1: Fig. S9, Additional file 3: Table S3.1) of Areas Under the Operating Characteristic Curve (AUCs), indicating that the predication models reached an excellent threshold value [47]. This indicates that the models employed in this study could accurately depict the relationships between species and climate and could be used to predict habitat suitability for 481 threatened medicinal plant species. The results of prediction analysis indicated that the current suitable habitat areas were mainly distributed in southern and south-western China, including Hengduan Mountains, eastern Yunnan, the boundary areas between Vietnam and China, Hainan Island, and Taiwan Island (Fig. 8A). However, the future suitable habitat areas were predicted to shift from southern China to northern China and would be mainly distributed in the southwest and southeast parts of China, including south-western and south-eastern Xizang, Hengduan Mountains, eastern Yunnan, Taiwan Island, north-eastern Guangdong, and western Fujian (Fig. 8B). On the whole, under the climate change scenarios, the suitable habitat areas in southern China will get greatly reduced, while the suitable habitat areas in the west and east parts of China will increase in the future (Fig. 8A, B).

The distribution patterns of suitable habitat areas in diversity hotspots indicated that 124 hotspot grid cells (83%) were completely covered by the current suitable habitat areas, which were mainly located in Hengduan Mountains, Bashan-Wushan Mountains, Nanling Mountains, and the boundary areas between Vietnam and China (Fig. 8C, Additional file 1: Fig. S10A). The remaining 26 hotspot grid cells (17%) were just partially covered by the current suitable habitat areas, and mainly distributed in the northern Tianshan Mountains, Qilian Mountains, Bashan-Wushan Mountains, and Wuling Mountains (Fig. 8C, Additional file 1: Fig. S10A). The statistics for future suitable habitat areas and hotspot grid cells indicated that 89 hotspot grid cells (59%) were completely covered by the future suitable habitat areas and were mainly located in Hengduan Mountains, Nanling Mountains, Mufu-Lushan Mountains, Tianmu Mountains, and Changbai Mountains (Fig. 8D, Additional file 1: Fig. S10B). A total of 61 hotspot grid cells (41%) were partially or completely located outside the future suitable habitat areas, which were mainly distributed in Tianshan Mountains, Qilian Mountains, Bashan-Wushan, the Xishuangbanna region, and the boundary areas between Vietnam and China (Fig. 8D, Additional file 1: Fig. S10B).

In total, 84 hotspot grid cells (56%) were distributed in long-term stable suitable habitat areas, which were mainly located in Hengduan Mountains, Nanling Mountains, Tianmu Mountains, and Changbai Mountains (Additional file 1: Fig. S10C). Among these hotspot grid cells, 16 were identified as national and provincial conservation gaps, and located in Hengduan Mountains (north-western Yunnan), Tianmu Mountains, and eastern Zhejiang (Fig. 8E, F, Additional file 1: Fig. S10D). Forty hotspot grid cells (27%) will face shrinkage or disappearance of suitable habitat areas in the future, mainly in the Bashan-Wushan Mountains, the Xishuangbanna region, and the boundary areas between Vietnam and China (Additional file 1: Fig. S10E). Among these 40 hotspot grid cells, 5 were identified as national and provincial conservation gaps, which were mainly located in Altai Mountains, Hengduan Mountains (western Sichuan), the Xishuangbanna region, and southern Guangdong (Fig. 8E, F, Additional file 1: Fig. S10F).

Further analysis of geographic distribution of suitable habitat areas and national and provincial nature reserves showed that there are significant apparent shifts of suitable habitat areas in spatial distribution patterns compared with distribution patterns of nature reserves. For example, with the diminishing of suitable habitat areas in southern Yunnan, south-western Guangxi, southern Guangdong, and Qingling Mountains, nature reserves located in these areas will confront the disappearance of suitable habitat areas in the future. However, some areas will encounter a remarkably improving quality of suitable habitat areas, such as south-western China (including central and eastern Xizang, western and eastern Sichuan), north-western China (e.g., central, and southern Xinjiang), north-eastern China (for example Changbai Mountains), and south-eastern China (especially in northern Guangdong, western Fujian, and south-western Zhejiang) (Additional file 1: Fig. S11, S12).

## Discussion

In this study, we present integrated distribution patterns of all medicinal plants in China based on a high-resolution network analysis, employing large amounts of precise species occurrence data and two different algorithms. This sheds new light on understanding the hotspot areas, conservation effectiveness and conservation gaps of Chinese medicinal plants, and the risk of shift and loss of suitable habitat areas under climate change.

### The significance of hotspot areas for conservation priority

The diversity hotspots identified in this study were based on the principles of species richness and species irreplaceability, which could help to guide the limited available conservation resources to those regions that need it most [8, 20, 48]. The hotspot grid cells identified here are of high value in conservation priority for which only accounted for 5% of the total distribution area but harbored a larger proportion (96%) of all medicinal plant species, notably national protected medicinal plants (100%) and CITES listed medicinal plants (99%, Fig. 7, Table 1). Although national and provincial nature reserves covered more land areas (34% and 37%) than diversity hotspots, they had no obvious advantage in containing species (Fig. 7, Table 1). Moreover, the diversity hotspots identified in this study not only covered all traditional hotspots areas, such as the Hengduan Mountains, Nanling Mountains, and Bashan-Wushan Mountains [11, 49–51], but also included some precise new hotspots, such as the Mufu-Lushan Mountains, identified by the top 5% richness algorithm, and the Tianshan-Altai Mountains and Changbai Mountains identified by complementary algorithm (Fig. 5A).

The distribution pattern of diversity hotspots we identified is basically coincides with the hotspots based on the county-level distribution data for 535 threatened medicinal plant species [2]. However, we identified more hotspot grid cells in the Hengduan Mountains and found new hotspot areas in the Nanling Mountains and Tianmu Mountains, which may reflect the advantages of high-resolution distribution data. Moreover, our diversity hotspots were also supported by the distribution pattern of 3150 Chinese endemic medicinal plant species, based on the provincial distribution data, which indicated that south-western China possessed the highest species diversity of endemic medicinal plant species [22]. Our analysis also showed that the national nature reserve network has high conservation effectiveness (97%) of medicinal plant species, which is consistent with evaluations that focused on central and northern China [24, 25].

Traditionally, biodiversity hotspots have been identified only based on species richness [8, 10, 49, 50]. Compared to the top 5% richness algorithm, the integrated hotspots of complementary algorithm in this study possessed great advantages in the species ratio of all five groups, especially in threatened medicinal plant species (the top 5% richness algorithm vs. the complementary algorithm: 85%/96%), and national protected medicinal plant species (89%/100%, Table 1), indicating that the complementary algorithm greatly improves the representativeness and accuracy of hotspot delimitation in the comprehensive analysis of both algorithms. Furthermore, correlation analysis also showed that the coherence between the distribution patterns of the two algorithms was quite low, which indicates that species complementarity represents another aspect of species’ diversity differing from species richness. Our findings confirmed previous studies indicating that the comprehensive consideration of multiple methods can yield more reasonable hotspots and thus better scientifically based [19]. In total, the hotspot grid cells obtained through comprehensive methods and high-resolution distribution data in our study are critical for the conservation of medicinal plants and bringing hotspot grid cells into the priority areas could effectively improve conservation effectiveness of this resource.

### Optimization of the network of nature reserves for medicinal plants

Based on conservation efficiency, the current conservation networks, both NNR and PNR, play a great role in protecting medicinal plants. However, 25 hotspot grid cells (16.7%) were still outside the conservation network due to the incongruent distribution pattern of nature reserves and hotspot grid cells. Therefore, priority should be given to these national and provincial conservation gaps in Tianshan-Altai Mountains and Hengduan Mountains when establishing new nature reserves or expanding the existing nature reserves (Fig. 5D). In addition, we noticed that the provincial nature reserves play an important role in the conservation of medicinal plants in China. On the basis of national nature reserves, we found that the number of conservation gaps was clearly reduced (from 49 fall to 25) and the conservation effectiveness of medicinal plant species was increased (from 97 up to 99%) when considering the effectiveness of national and provincial nature reserves together (Table 1). Usually, the national nature reserves are considered to be managed more rigorously and receiving more manpower and financial support than the provincial nature reserves [52]. Therefore, it is necessary to pay more attention to the important role of provincial nature reserves, and the management or investment of provincial nature reserves should also be strengthened when developing the conservation plan to conserve medicinal plants in the future.

Among a total of 9756 medicinal plant species included in this study, only 9% had a distribution range covering less than five grid cells (about 1.51 × 10^4^ km^2^), while more than 66% of medicinal plants had a distribution range of more than twenty grid cells (6.05 × 10^4^ km^2^). This indicates that most medicinal plants are widespread species. According to previous studies widespread species are more likely to be protected than the narrow-ranged species [53], which may explain the high conservation effectiveness of nature reserves for medicinal plants. Nevertheless, in the long run, the protection of medicinal plants still needs to be strengthened, especially considering the huge demand for medicinal plants [1, 2, 54]. The high proportion of widespread species recorded in medicinal plants also reflects that most of the medicinal plants are highly accessible to the public [55, 56], which indicates that by improving the public awareness of biodiversity conservation of medicinal plants we can strengthen the protection of this resource.

Other strategies should also be considered to better conserve medicinal plants. We noticed that considerable hotspot grid cells were located along provincial boundaries, exemplified by the hotspot grid cells located in the Bashan-Wushan Mountains, Nanling Mountains, Mufu-Lushan Mountains, etc. Currently, in order to facilitate management, nature reserves do not cross administrative boundaries in China, which may reduce the connectivity between habitats and the conservation effectiveness of hotspots of medicinal plants [57, 58]. Therefore, we suggest removing this limitation to better conserve medicinal plants, including expanding current nature reserves, or establishing new cross regional national parks to conserve these hotspot grid cells. Compared with the establishment of large-scale nature reserves or national parks, the establishment of plant micro-reserves for threatened medicinal plants has many apparent advantages, including lower cost, fast construction, strong pertinence, and also improving the public awareness of biodiversity conservation of medicinal plants among local residents and even the society [59–61]. Therefore, plant micro-reserves should be established in the concentration areas of threatened medicinal plants that are under serious threat to conserve their original habitats and genetic resources. At the same time, other methods, such as in vitro conservation, seed banks, DNA barcoding, in situ cultivate, etc., can be used to conserve the wild germplasm resources of these threatened medicinal plants.

### Conservation priority of medicinal plants in suitable habitat areas

The results of our study showed that the future suitable habitat areas of medicinal plants will shift northward under a global warming scenario (Fig. 8A, B), which is consistent with previous studies [29, 41, 62]. Although some new suitable habitat areas will occur in the south-eastern Xizang, western Fujian, and north-eastern Guangdong, there will be an obvious decline of suitable habitat areas in the boundary areas between Vietnam and China, Bashan-Wushan, and the Xishuangbanna region which currently harbor a particular richness of medicinal plants. Therefore, climate change will pose a great challenge to conservation of these resources. Moreover, given that the growth of high-quality medicinal plants is highly dependent on coaction of excellent genetic genes and specific growth environment [6], the new suitable habitat areas may not meet the needs of medicinal plants to grow, so more attention needs to be given to the long-term stable suitable habitat areas.

The prediction of suitable habitat areas can provide a basis for the optimization and re-layout of the current conservation networks. This study indicated that many hotspot grid cells distributed in Hengduan Mountains, Nanling Mountains, Tianmu Mountains, and Changbai Mountains were located in long-term stable suitable habitat areas, indicating that conservation efforts in these hotspot grid cells should be strengthened to achieve the long-term conservation of medicinal plants. The hotspot grid cells located in Bashan-Wushan Mountains, the Xishuangbanna region, and the boundary areas between Vietnam and China will face a great challenge caused by climate change in future. It is necessary to conduct periodic resource surveys and threat assessments for medicinal plant resources to dynamically monitor the changes of medicinal plant distribution in these hotspot grid cells. Other conservation actions to strengthen the management of medicinal plants are needed, including establishing specific nature reserves for precious and endangered medicinal plants, strengthening professional skills training of relevant staff, and increasing the publicity of popular science to improve people’s reasonable understanding of the medicinal value of medicinal plants. When necessary, ex situ and germplasm conservation techniques need to be used to preserve the genetic resources of medicinal plants in these hotspot grid cells. In addition to the hotspot grid cells mentioned above, we also suggest that the layout of future nature reserves could be optimized by considering the transfer of suitable habitat areas (including the loss and increase of suitable habitat areas) and the current distribution of nature reserves.

### Illustration for the data integrity

The distribution data used in this study were accessed from the Chinese Virtual Herbarium (CVH), which integrates specimen records of hundreds of major herbaria in China and possesses more than 8.6 million specimen records. At present, it is the most comprehensive data and the best choice to explore the distribution patterns of medicinal plants in China. Many previous studies have shown that the distribution data of CVH basically cover the whole of China, and using these distribution data to explore the distribution pattern of different plant groups could obtain relatively consistent, realistic, and reliable results, for Chinese angiosperms, higher plants in general, endemic plants and relict plants, etc. [13, 35, 63–65]. Therefore, we believe selecting the most comprehensive data sets at this stage to explore the distribution pattern of medicinal plants is reasonable and feasible, and the deviation of the data is very limited.

## Conclusions

Based on big data and multiple indicators, 150 hotspot grid cells covering only 5% of the total distribution area of medicinal plants, but harboring 96% of all medicinal plants, were identified. These hotspots not only covered all traditional hotspots but also included three new hotspot areas of medicinal plants. This study fully confirmed the importance of comprehensive consideration of multiple indicators and methods in identifying biodiversity hotspots. The fact that most medicinal plants were widespread species led to a high conservation effectiveness of current conservation networks for medicinal plants. But considering the huge demand for medicinal plants, and existing conservation gaps especially in the Tianshan-Altai Mountains and Hengduan Mountains, priority should be given to these gaps when establishing new nature reserves or enlarge existing nature reserves. Other measures, such as removing the limitation of nature reserves in Bashan-Wushan Mountains, Nanling Mountains, Mufu-Lushan Mountains, and the establishment of plant micro-reserves for threatened medicinal plants, should also be considered. The prediction of suitable habitat areas under climate change showed that the Hengduan Mountains, Nanling Mountains, Tianmu Mountains, and Changbai Mountains can serve as long-term conservation priority areas due to their very stable habitat suitability and high diversity of medicinal plants.

## Methods

### Checklist and database of occurrence data of medicinal plant species

A checklist of Chinese medicinal plants was compiled using the existing literature concerning traditional Chinese medicine in China [66–68] and was further combined with the latest field survey data [69]. This checklist of medicinal plants covers all medicinal plants in China, including the three main medicine systems in China together (Han Chinese medicine, folk medicine, and minority medicine). Then the checklist was updated according to the Species catalog of China to reject synonyms and uncertain names [70]. All subspecies or varieties were retained and treated as individual entries, whereas cultivated and alien species were excluded. According to the checklist, occurrence data of specimens were accessed and applied from Chinese Virtual Herbarium [71]. Specimen records lacking detailed locality information were excluded. We then geo-referenced these occurrence records with accurate coordinate information according to Chinese gazetteers. After removing invalid data, we obtained 634,314 georeferenced points for the analysis of the distribution patterns of medicinal plants, including 9756 species, belonging to 2087 genera and 285 families.

To identify biodiversity hotspots for conservation priority, we synthesized species richness distribution patterns of five groups, just all medicinal plants, endemic medicinal plants, threatened medicinal plants, CITES listed medicinal plants, and national protected medicinal plants. Medicinal plants in the checklist included 620 (6.4%) threatened medicinal plant species [42], 3205 (32.9%) endemic medicinal plant species [51, 72], 344 (3.5%) CITES listed medicinal plant species [73], and 76 (0.8%) national protected medicinal plant species [74]. The threatened medicinal plants were further classified into three categories, namely VU (Vulnerable), EN (Endangered), and CR (Critically endangered) [42]. The list of species under each of these categories is presented in Additional file 3: Table S3.2.

### Identification of distribution patterns for species richness and hotspots

To visualize the distribution patterns and identify hotspots of medicinal plants, the territory of China was divided into 4204 grid cells with 0.5 × 0.5° resolution using ArcGIS 10.0 (ESRI, Redlands, CA, USA), with each grid covering an area of approximately 3025 km^2^. The top 5% richness algorithm and the complementary algorithm were employed to analyze hotspot distribution patterns of medicinal plants [2, 11, 18].

The top 5% richness algorithm defines the hotspots as the top 5% area of the study area with the highest species richness. In order to comprehensively consider the five groups defined above, we converted the species number of each group in each grid cell into a ratio of the species number to the total number in their corresponding group. Next, we aggregated the five ratios to obtain an index to measure the species richness in each grid cell (Additional file 3: Table S3.3).

In contrast, the complementary algorithm identifies the hotspots by selecting the minimum number of grid cells that covers all plant species [2, 18]. For the complementary algorithm, we first selected the grid cell with the highest species richness, and all species that occur in this grid cell are excluded from further consideration. We then selected the grid cells with the highest number of remaining species and continued this process iteratively until all species were included in the selected grid cells. We calculated the ratio for all five groups following the treatment of the top 5% richness algorithm. We summed these five ratios to evaluate the species complementarity of each grid cell (Additional file 3: Table S3.4).

A total of 3047 grid cells were filled with occurrence data, and the top 150 grid cells with highest species richness were selected as integrated hotspots of the top 5% richness algorithm (Additional file 3: Table S3.3). In order to compare to the results of the top 5% richness algorithm, we also selected the top 150 grid cells with highest species complementarity as integrated hotspots of the complementary algorithm. For the complementary algorithm, 151 grid cells were selected because the order 151 and 150 received same value (Additional file 3: Table S3.4). Finally, we combined the integrated hotspots of the two algorithms, calculating the sum of orders of each hotspot grid cell in the respective algorithms. We sorted the sum of two orders or one order and treated the 71 grid cells identified by both algorithms as first-class hotspots. We then sorted the remaining grid cells identified by only one algorithm and selected the top 79 grid cells as second-class hotspots (Additional file 3: Table S3.5). The resulting 150 hotspot grid cells were treated as final diversity hotspots of medicinal plants.

### Correlation analysis on distribution patterns of medicinal plants

To detect the relationships and congruence among the different distribution patterns of medicinal plants, we employed the “corrplot package” in software R (version 4.0.2) to conduct a correlation analysis on distribution patterns of species richness and species complementarity of all medicinal plants, endemic medicinal plants, threatened medicinal plants, CITES listed medicinal plants, and national protected medicinal plants [75]. We first normalized the variables and the grid cells absence value with zero (value 0) to make the procedure run smooth during correlation analysis (Additional file 3: Table S3.6). For correlation analysis on distribution patterns, we calculated Pearson’s correlation coefficient to measure the related degree of different distribution patterns by sorting of |*r*| value into seven classes: perfect correlation (|*r*|=1), very strong correlation (0.8≤|*r*|<1), strong correlation (0.6≤|*r*|<0.8), moderate correlation (0.4≤|*r*|<0.6), weak correlation (0.2≤|*r*|<0.4), very weak correlation (0<|*r*|<0.2), and no correlation (*r* = 0) [76].

### Conservation effectiveness and gaps analysis

The conservation effectiveness and conservation gaps were evaluated based on the distribution patterns of hotspot grid cells and the current nature reserves. Up to date, China has established more than 2700 nature reserves, accounting for nearly 15% of its total land [77]. Many studies have shown that the network of nature reserves in China plays a significant role in conserving biodiversity, natural landscapes, and ecosystem services [78, 79]. Nature reserves in China are usually classified into four categories (from the highest to the lowest: national, provincial, municipal, and county), managed by different levels of government. Here we only selected the national and provincial nature reserves, mainly because they represent the majority of the nature reserves network of China and are strictly managed, while the municipal and county nature reserves are often not well maintained due to insufficient funds [21, 79].

First we compiled a geo-document of current nature reserves based on the data obtained from the World Database on Protected Areas [80] and updated them according to the relevant documents of nature reserves issued by the government up to 2018 [81]. This conservation network was composed of 464 national nature reserves and 806 provincial nature reserves. We then overlapped the layers of nature reserves on the layer of final hotspot grid cells and counted the hotspot grid cells covered by national nature reserves or provincial nature reserves as conservation effectiveness, and identified conservation gaps by marking the of hotspot grid cells not covered by national nature reserves or provincial nature reserves.

### Statistics on composition of medicinal plants

In order to evaluate the conservation effectiveness and gaps for hotspot areas and all grid cells with distribution data more synthetically, we elucidated the inner comprising relationships of different groups by using the “circlize” [82] and “tidyverse” [83] packages in software R (version 4.0.2), which could present the proportion of the hotspot grid cells and different groups in the form of a chord diagram and circular barplot. In order to avoid overlapping of different groups and better visualizing the statistic results of the relationships of different groups, we divided all species into three groups according to conservation priority, i.e., threatened species, endemic species (excluding threatened species), and the remaining species (excluding threatened and endemic species).

### Prediction of suitable habitat areas for threatened medicinal plants

Most threatened species are sensitive to environmental change, and thus can reflect the impact of climate change on biodiversity [33, 84]. Therefore, we attempted to use MaxEnt (version 3.3) to predict the current and future suitable habitat areas for threatened medicinal plants, which has been shown to be a suitable method for modeling species distribution with presence-only data [33, 85, 86]. The prediction was performed on 481 threatened species with at least 5 distribution points. Threatened species with less than 5 distribution points were excluded due to the low predictive ability of MaxEnt for these species [35, 87] (Additional file 3: Table S3.1). Nineteen bioclimatic variables (Additional file 3: Table S3.7) with 5 arc min resolution for the current climate data (1950-2000, WorldClim, version 1.3) and future climate data (2100, 2xCO2 climate conditions, CCM3 mode) were downloaded from DIVA-GIS database [88], and two other environmental variables (topographic variables and elevation) were downloaded from the USGS’s Hydro-1K database [89]. To minimize the impact of multi-collinearity and over-fitting on the stability and quality of models, 19 bioclimatic variables and two environmental variables were analyzed by principal component analysis and the Pearson’s correlation test and one of the two variables was removed when a correlation coefficient > |0.70| was obtained. Finally, eight environmental variables, including BIO1, BIO5, BIO6, BIO7, BIO12, BIO13, BIO16, and elevation (Additional file 3: Table S3.7) were selected to predict the current and future suitable habitat areas.

We used two methods of replication to construct the MaxEnt models. For the species with 5–29 occurrence records, we used jackknife and ran the same number of replicates with that of occurrence points [35, 87]. For the species with more than 30 occurrence records, we applied a cross-validation approach and ran 10 replicates to obtain more robust modeling results [35]. Default values were used for all the remaining parameters. The suitable habitat areas were then predicted for threatened medicinal plants under current and future climate scenarios. After predicting suitable habitat areas for each threatened medicinal plant, we used ArcGIS 10.0 to extract the value of habitat suitability for each species in different grid cells. In this process, we first rejected the suitable habitat areas with suitability value of less than 0.75 for each species, because suitability values greater than 0.6 were considered to be high potential [33, 90]. In the remaining grid cells, the suitability values of different species in the same grid were superposed, and the final sum was considered as the final suitability value of the grid. We then used ArcGIS 10.0 to visualize the current and future suitable habitat areas. Finally, we overlapped suitable habitat areas with hotspot grid cells, conservation gaps, and nature reserves to evaluate the impacts of climate change on diversity conservation of medicinal plants.

## Supplementary Information

**Additional file 1: Fig. S1**. Dragon Boat Festival wild medicinal plants market in Gongcheng, Guangxi China. **Fig. S2**. Map of the main mountains range in China. **Fig. S3**. Administrative division map of China (including the distribution of neighboring countries) (http://bzdt.ch.mnr.gov.cn/index.html). **Fig. S4**. The top 151 grids with highest species richness of all medicinal plants (AMPs_SR). **Fig. S5**. The top 152 grids with highest species richness of endemic medicinal plants (EMPs_SR). **Fig. S6**. The top 157 grids with highest species richness of threatened medicinal plants (TMPs_SR). **Fig. S7**. The top 156 grids with highest species richness of CITES listed medicinal plants (CMPs_SR). **Fig. S8**. The top 145 grids with highest species richness of national protected medicinal plants (NPMPs_SR). **Fig. S9**. The statistics of Areas under the Operating Characteristic Curve (AUC) of each threatened medicinal plants in MaxEnt. **Fig. S10**. Superposition of current and future suitable habitat areas with diversity hotspots and conservation gaps. **Fig. S11**. Superposition of current suitable habitat areas (current SHAs) with national and provincial nature reserves (NNRs-PNRs). **Fig. S12**. Superposition of future suitable habitat areas (future SHAs) with national and provincial nature reserves (NNRs-PNRs).

**Additional file 2: Table S2.1**. Hotspot areas of medicinal plants in China. **Table S2.2**. Species composition of different groups in China, diversity hotspots, conservation effectiveness and gaps for diversity hotspots, and grid cells covered by nature reserves focusing on the whole country.

**Additional file 3: Table S3.1**. The threatened species used for the predication of potential suitable habitat areas and their Areas under the Operating Characteristic Curve (AUC) in MaxEnt. **Table S3.2**. The list of total, endemic (China), threatened, CITES listed and national protected species of medicinal plants. **Table S3.3**. The species richness of each grid cell and the integrated hotspots of the top 5% richness algorithm. **Table S3.4**. The species number of each grid cell under complementary algorithm and the integrated hotspots of the complementary algorithm. **Table S3.5**. The diversity hotspot grids obtained from the integrated hotspots of both “top 5% richness algorithm” and “complementary algorithm”. **Table S3.6**. Correlation analysis of the distribution pattern of top 5% richness algorithm(R) and complementary algorithm(C) of all medicinal plants (All), endemic medicinal plants (EN), threatened medicinal plants (TH), CITES listed medicinal plants (CI) and national protected medicinal plants (NP). **Table S3.7**. The seven bioclimatic variables marked in red out of nineteen ones were employed in prediction of suitable habitat areas.

## Data Availability

The list of medicinal plants is available from the monograph “Summary of Chinese Traditional Medicine Resources” (reference [66] in the manuscript). Distribution data are available through the Chinese Virtual Herbarium (http://www.cvh.ac.cn/). Other data that support the findings of this study are available in the supplementary information of this article.
