# Supplementary material for: Developing long-term conservation priority planning for medicinal plants in China by combining conservation status with diversity hotspot analyses and climate change prediction
Source: BMC Biol. 2022 Apr 21;20:89. doi: 10.1186/s12915-022-01285-4 (PMC9027417; doi:10.1186/s12915-022-01285-4)

**Additional File 1. Supplementary Figures**

**(Figures S1-S12)**

**Developing long-term conservation priority planning for medicinal plants in China by combining conservation status with diversity hotspot analyses and climate change prediction**

**Figure S1.** Dragon Boat Festival wild medicinal plants market in Gongcheng, Guangxi China, showing that wide medicinal plants are confronting with over collection and consumption by local people.

**
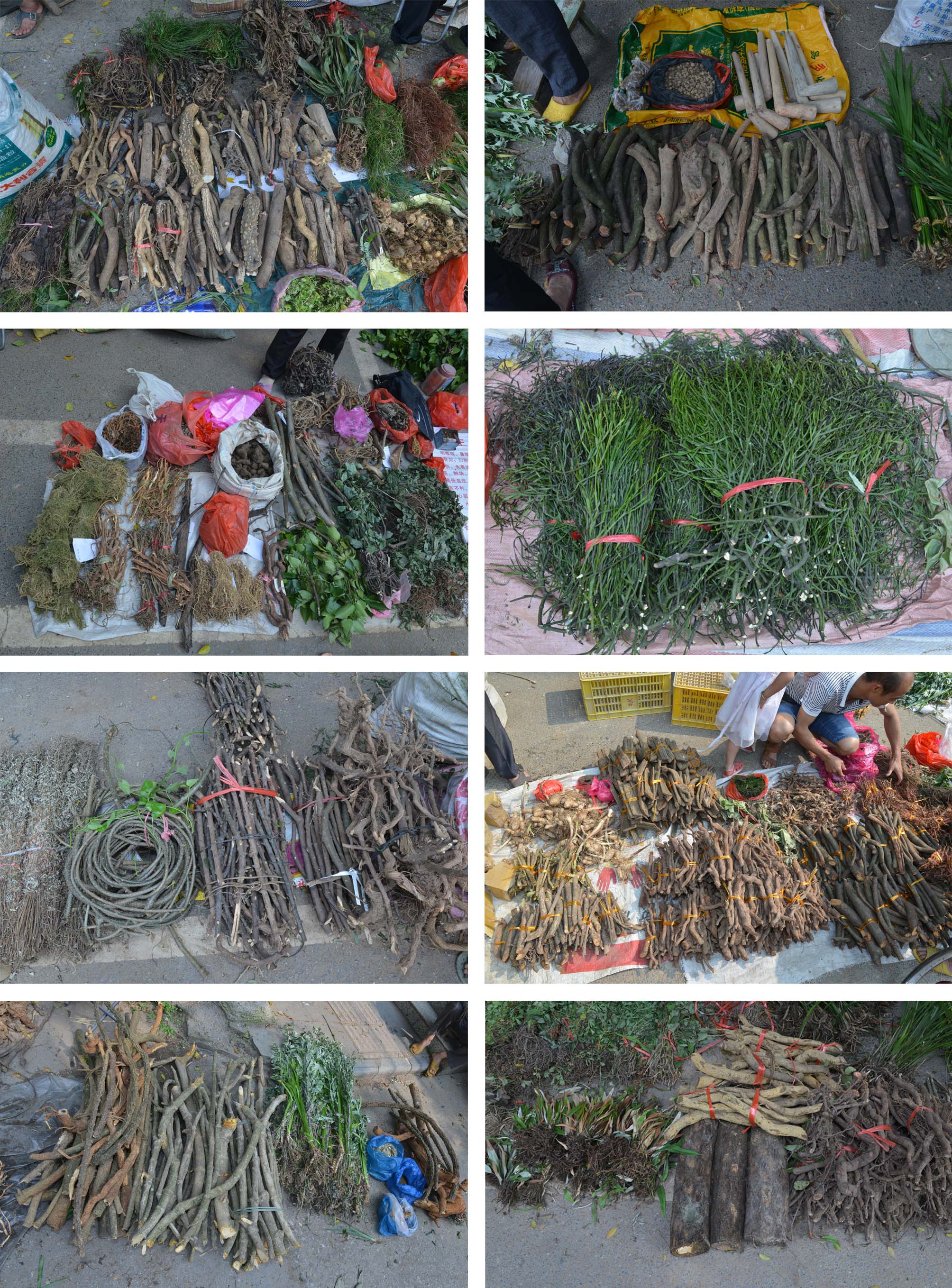
**

**Figure S2.** Map of the main mountains range in China: 1. Daxing'anling Mountains, 2. Xiaoxing'anling Mountains, 3. Changbai Mountains, 4. Zhangguangcailing Mountains, 5. Longgang Mountains, 6. Yinshan Mountains, 7. Yanshan Mountains, 8. Altai Mountains, 9. Tianshan Mountains, 10. Taihang Mountains, 11. Luliang Mountains, 12. Zhongtiao Mountains, 13. Helan Mountains, 14. Liupan Mountains, 15. Aerjin Mountains, 16. Qilian Mountains, 17. Kunlun Mountains, 18. Bayankala Mountains, 19. Qinling Mountains, 20. Daba Mountains, 21. Wushan Mountains, 22. Dabie Mountains, 23. Karakorum Mountains, 24. Danggula Mountains, 25. Gangdisi Mountains, 26. Nyainqntanglha Mountains, 27. Himalayas, 28. Hengduan Mountains, 29. Ailao Mountains, 30. Wuliang Mountains, 31. Wuling Mountains, 32. Xuefeng Mountains, 33. Dalou Mountains, 34. Mufu Mountains, 35. Lushan Mountains, 36. Luoxiao Mountains, 37. Tianmu Mountains, 38. Yandang Mountains, 39. Wuyi Mountains, 40. Nanling Mountains, 41. Dayao Mountains, 42. Yushan Mountains, 43. Wuzhi Mountains [46].


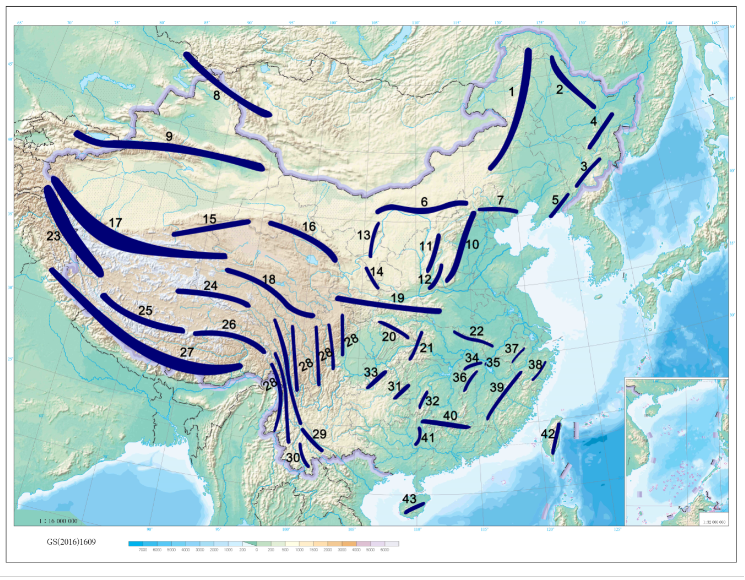


**Figure S3.** Administrative division map of China (including the distribution of neighboring countries) (http://bzdt.ch.mnr.gov.cn/index.html)

**
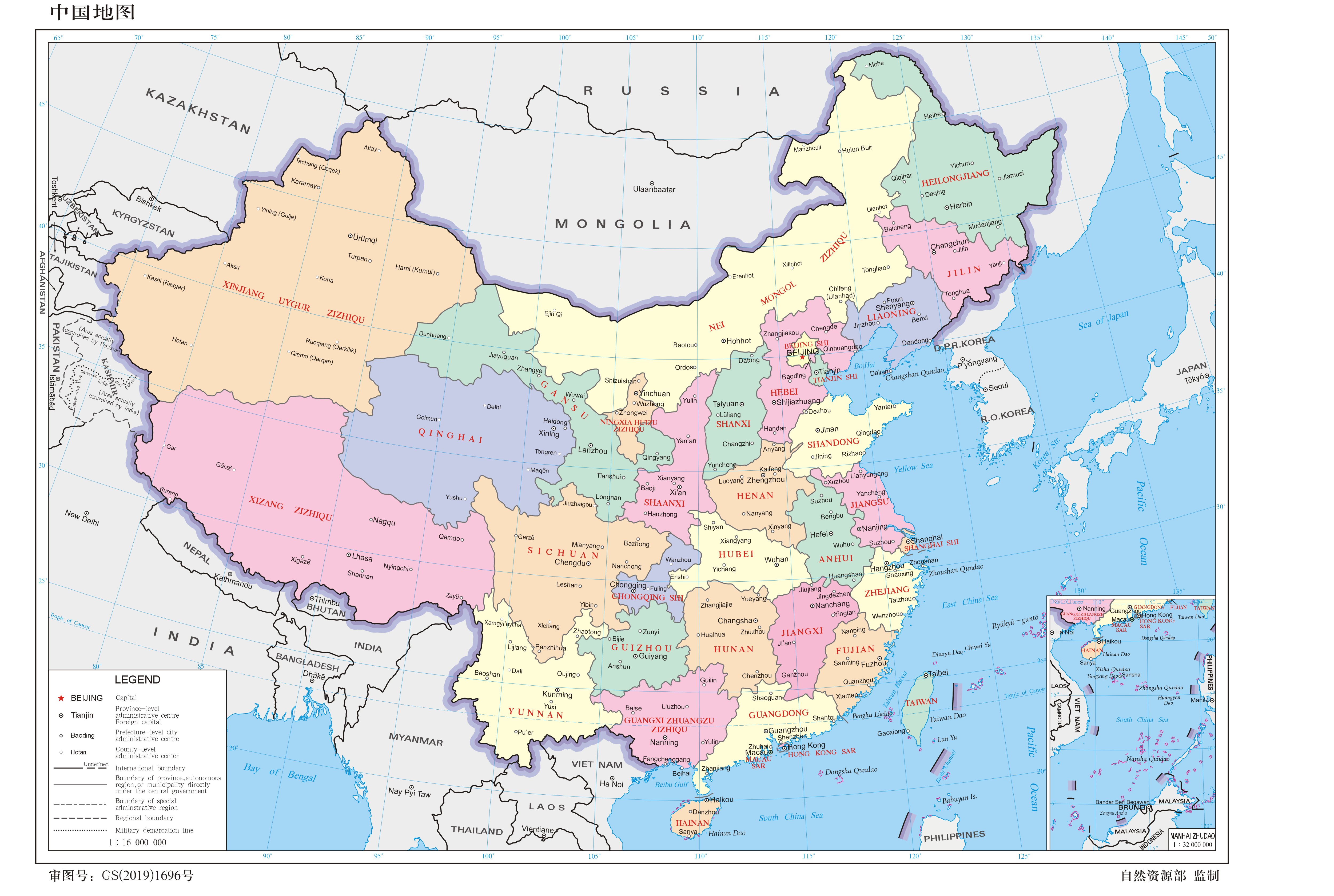
**

**Figure S4.** The top 151 grids with highest species richness of all medicinal plants (AMPs_SR).


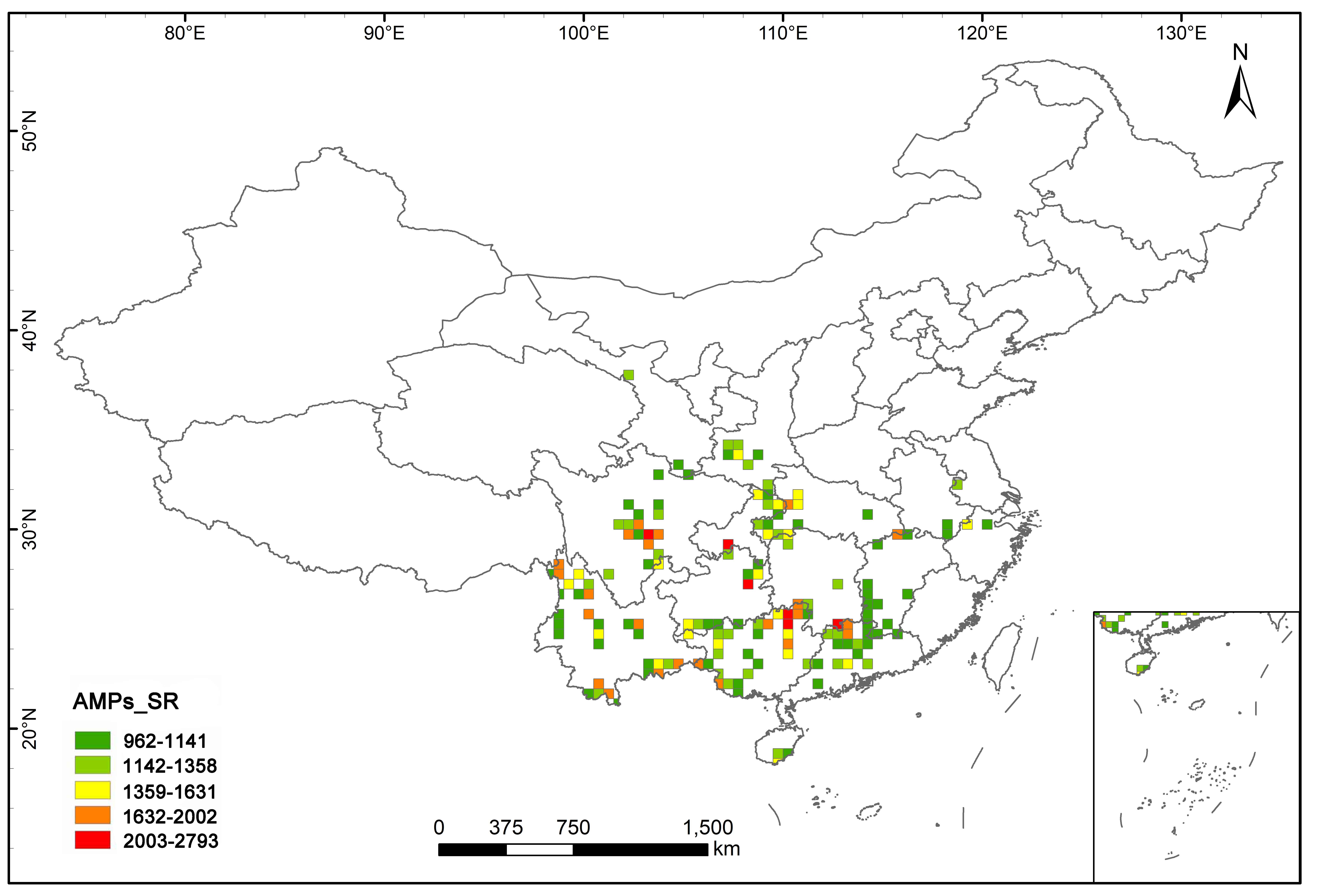


**Figure S5.** The top 152 grids with highest species richness of endemic medicinal plants (EMPs_SR).


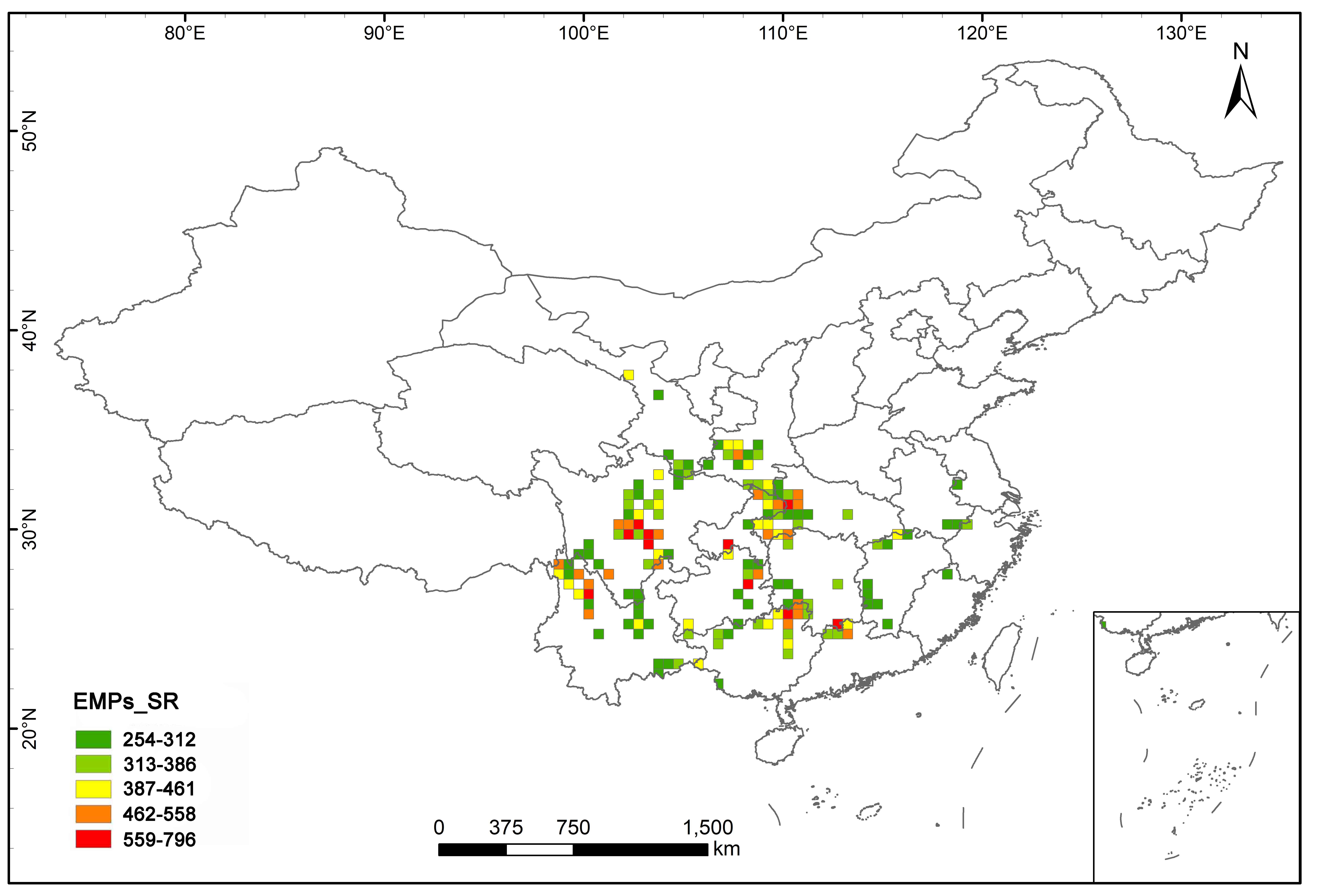


**Figure S6.** The top 157 grids with highest species richness of threatened medicinal plants (TMPs_SR).


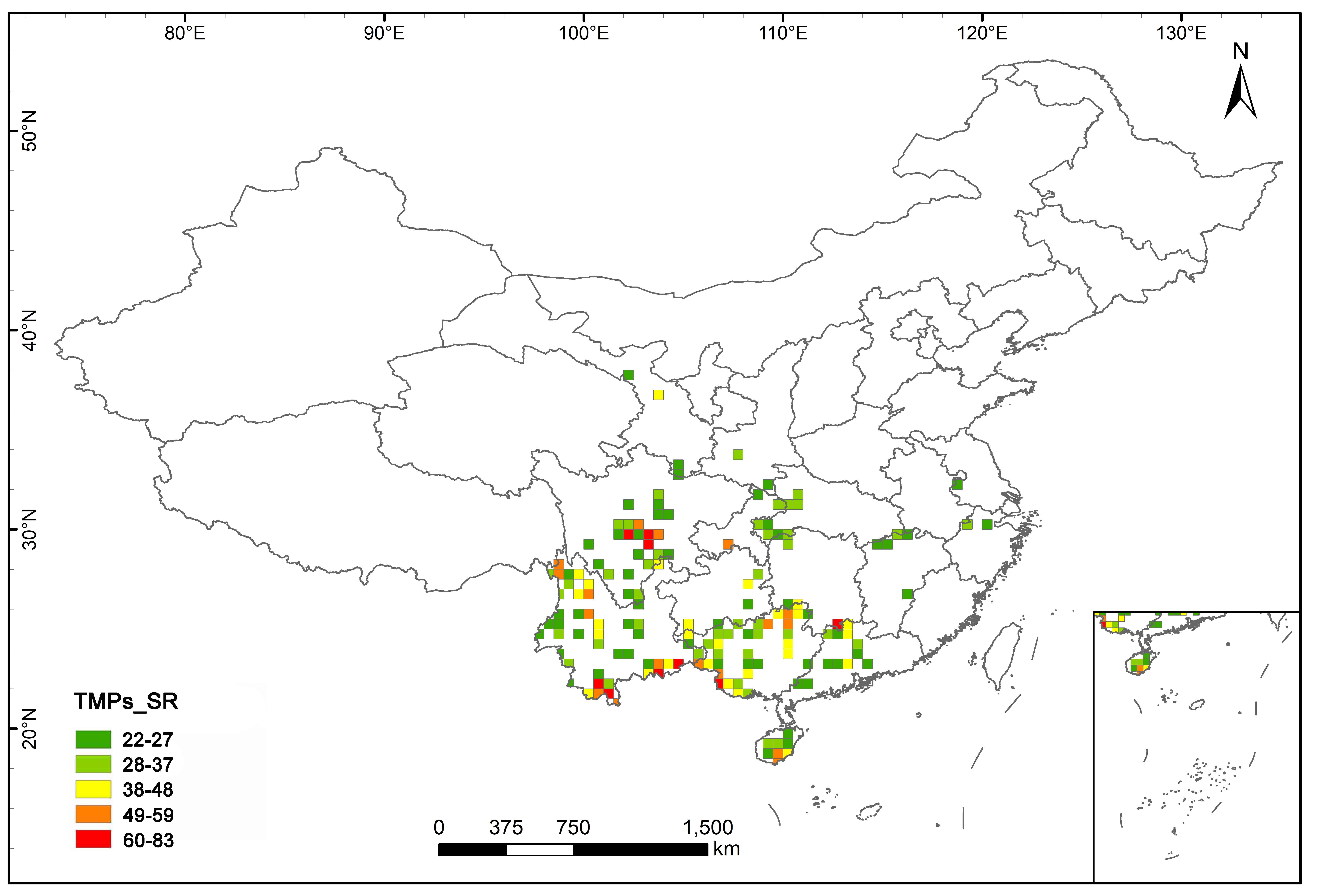


**Figure S7.** The top 156 grids with highest species richness of CITES listed medicinal plants (CMPs_SR).


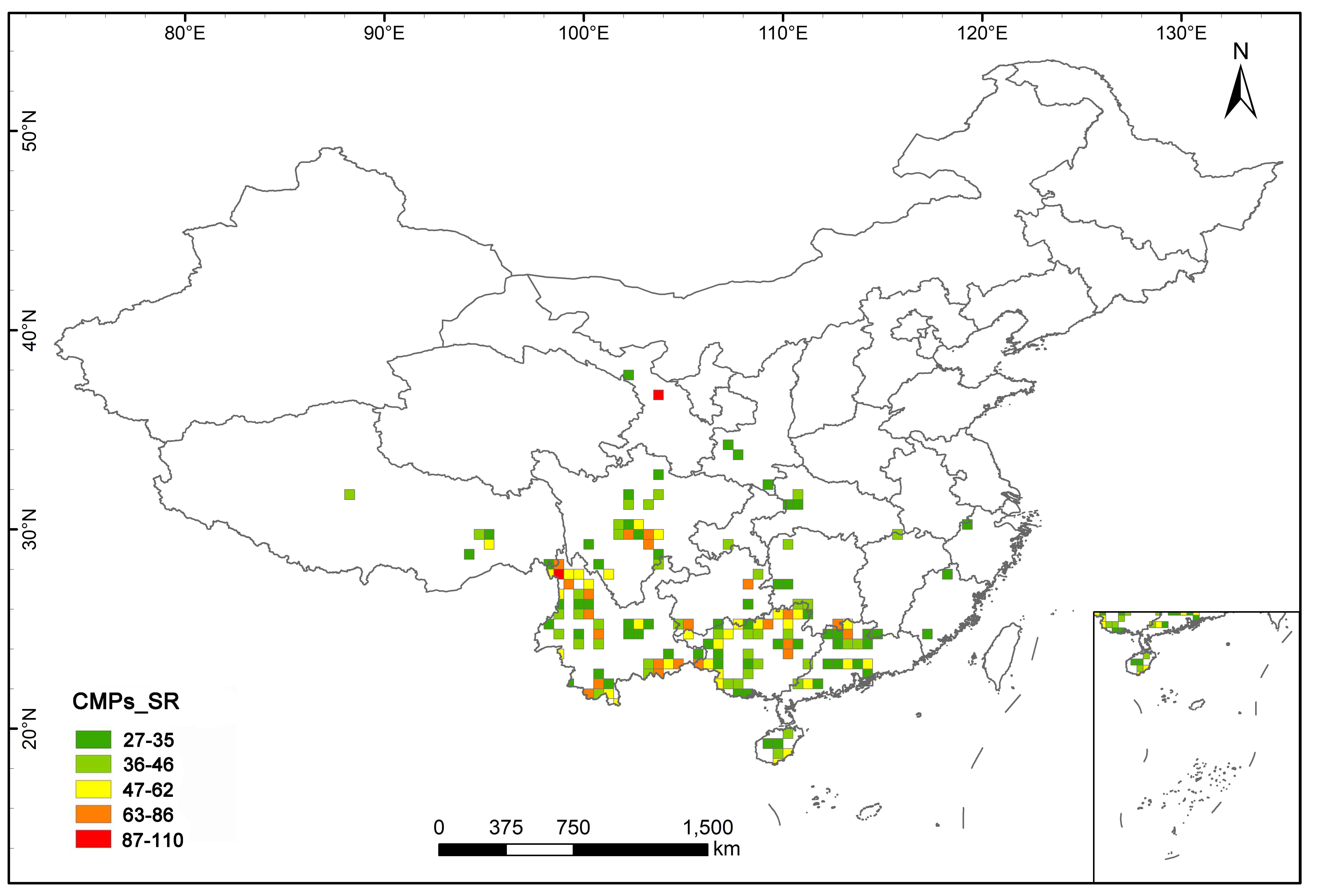


**Figure S8.** The top 145 grids with highest species richness of national protected medicinal plants (NPMPs_SR).


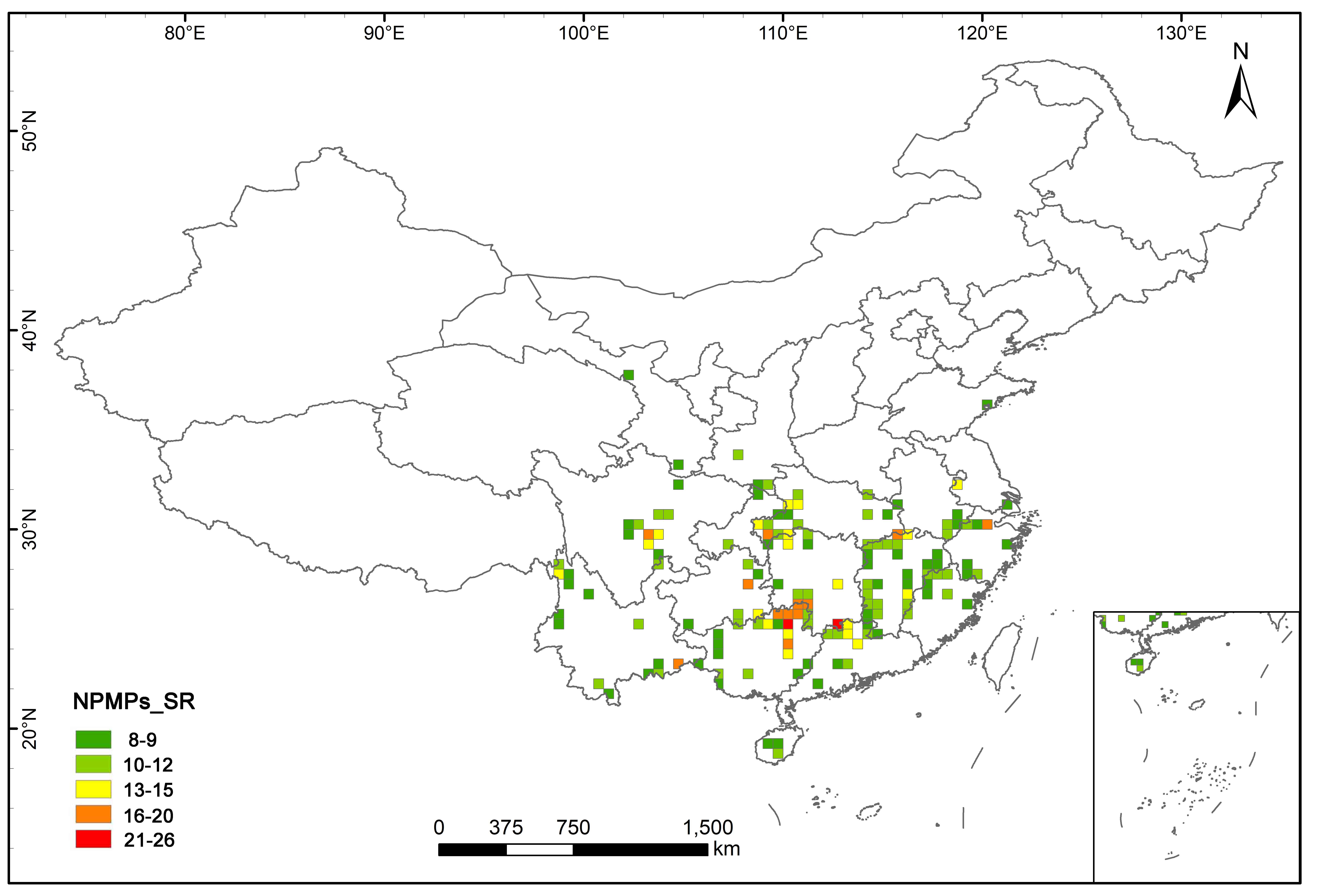


**Figure S9.** The statistics of Areas under the Operating Characteristic Curve (AUC) of each threatened medicinal plants in MaxEnt.


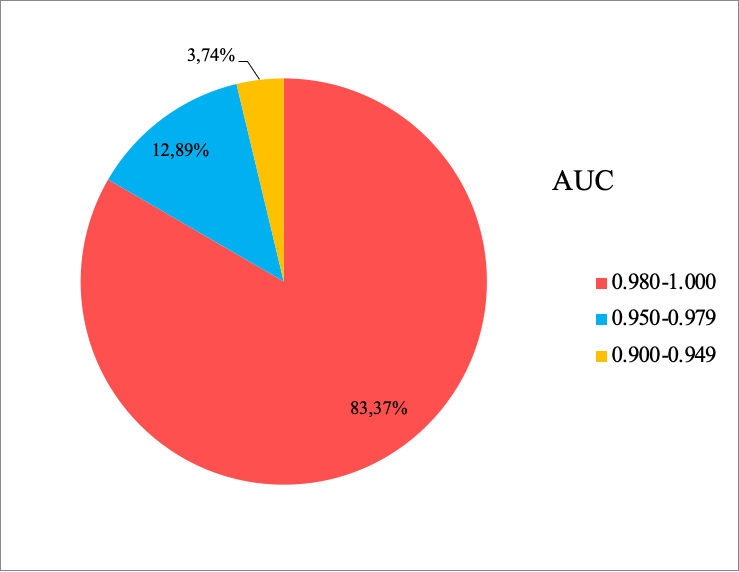


**Figure S10.** Superposition of current and future suitable habitat areas with diversity hotspots and conservation gaps. **A.** Superposition of current suitable habitat areas (SHAs) with hotspot grid cells (HGCs), with hotspot grid cells which covered completely by current suitable habitat areas in color bar (red, yellow, and green), which uncovered partially in blue. **B.** Superposition of future suitable habitat areas (SHAs) with hotspot grid cells (HGCs), with hotspot grid cells which covered completely by future suitable habitat areas in color bar (red, yellow, and green), which uncovered partially in blue, uncovered completely in purple. **C.** Diversity hotspots, with hotspot grid cells (HGCs) which located in stable suitable habitat areas (SHAs) in color bar (red, yellow and green), which located in unstable suitable habitat areas in blue. **D.** National and provincial conservation gaps (NNRs-PNRs gaps), with national and provincial conservation gaps which located in stable suitable habitat areas (SHAs) in black, which located in unstable suitable habitat areas in blue. **E.** Diversity hotspots, with hotspot grid cells (HGCs) facing the shrinking or disappearing of suitable habitat areas (SHAs) in future in blue. **F.** National and provincial conservation gaps (NNRs-PNRs gaps), with national and provincial conservation gaps facing the shrinking or disappearing of suitable habitat areas (SHAs) in future in blue.


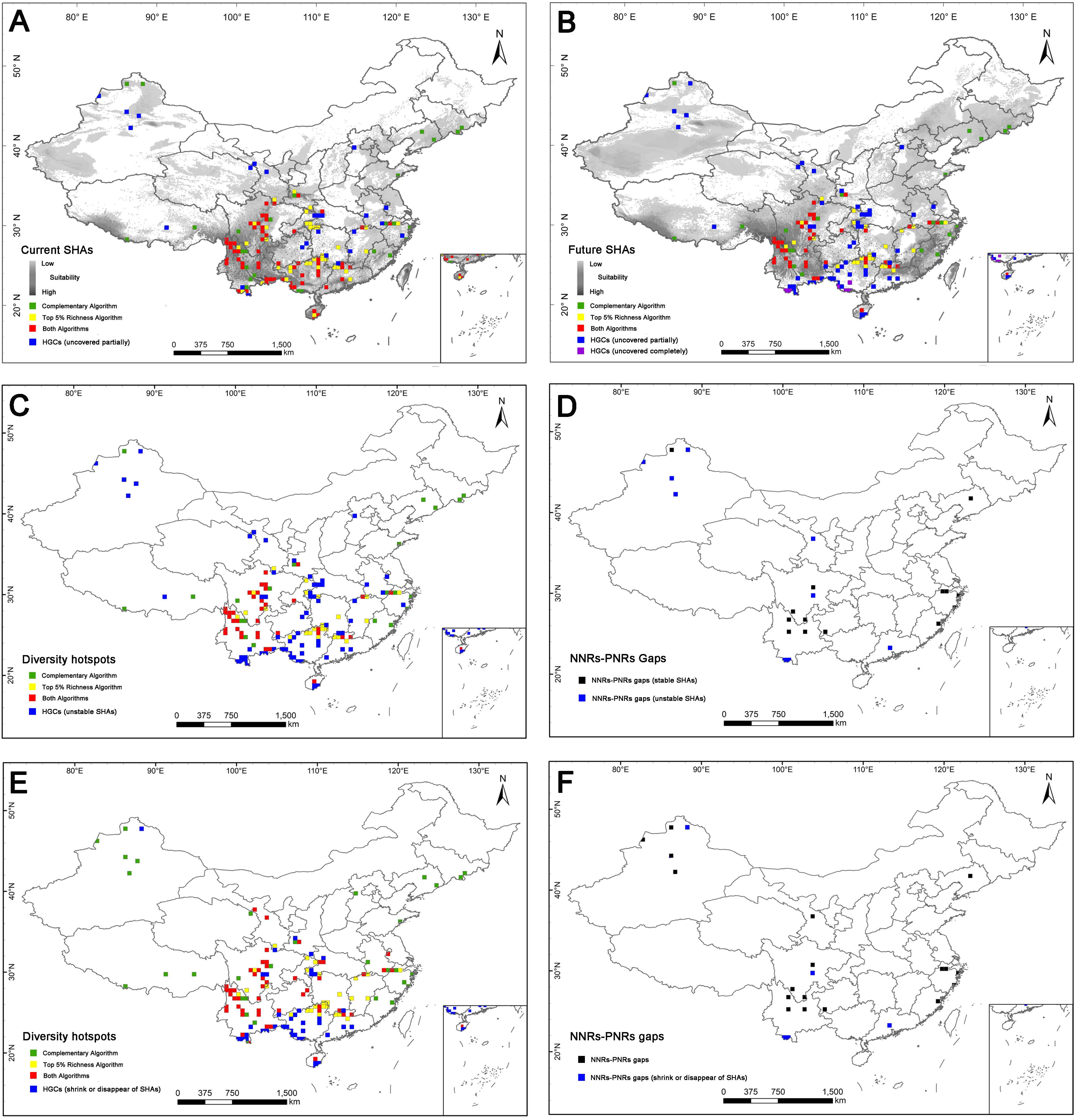


**Figure S11.** Superposition of current suitable habitat areas (current SHAs) with national and provincial nature reserves (NNRs-PNRs).


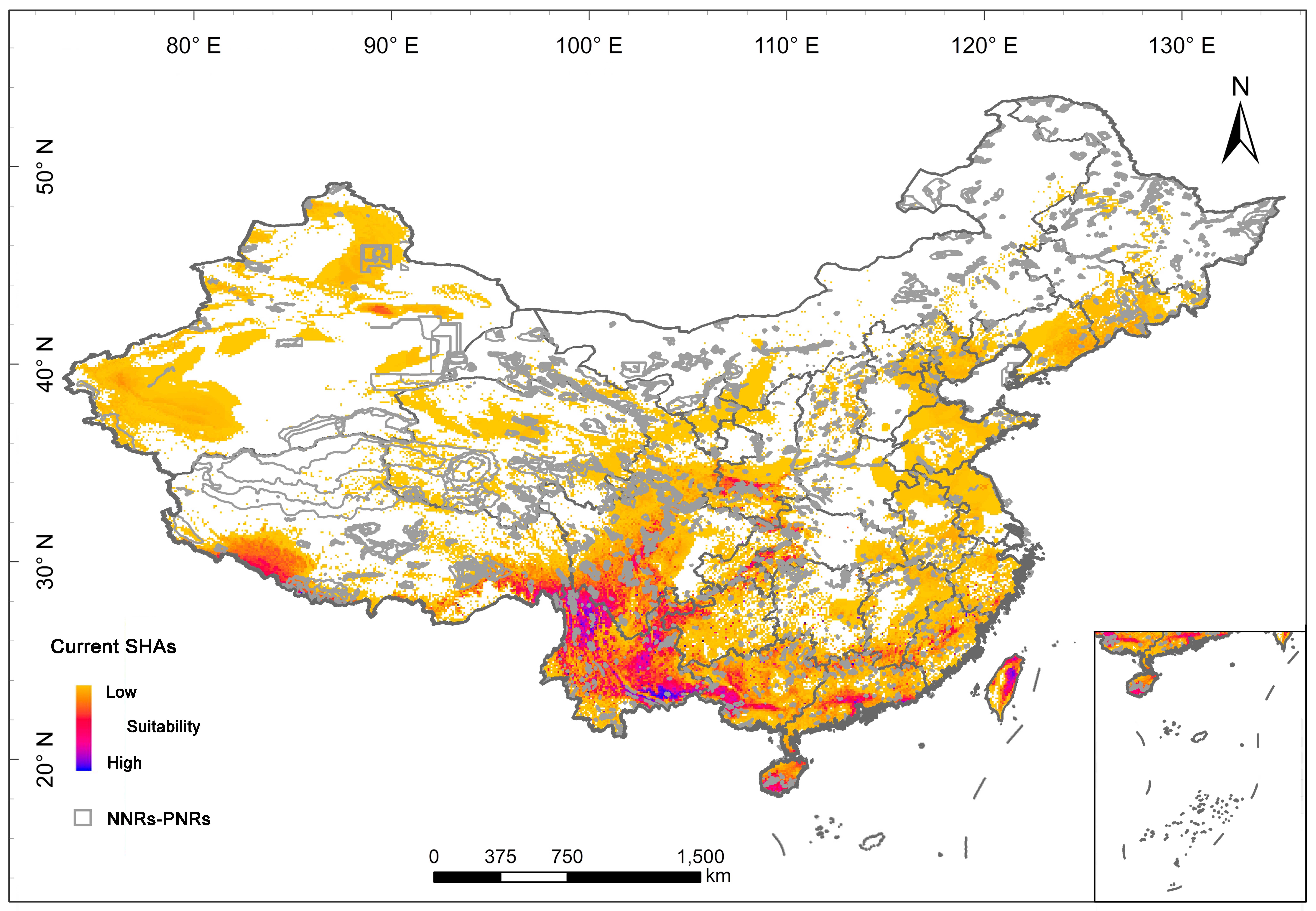


**Figure S12.** Superposition of future suitable habitat areas (future SHAs) with national and provincial nature reserves (NNRs-PNRs).


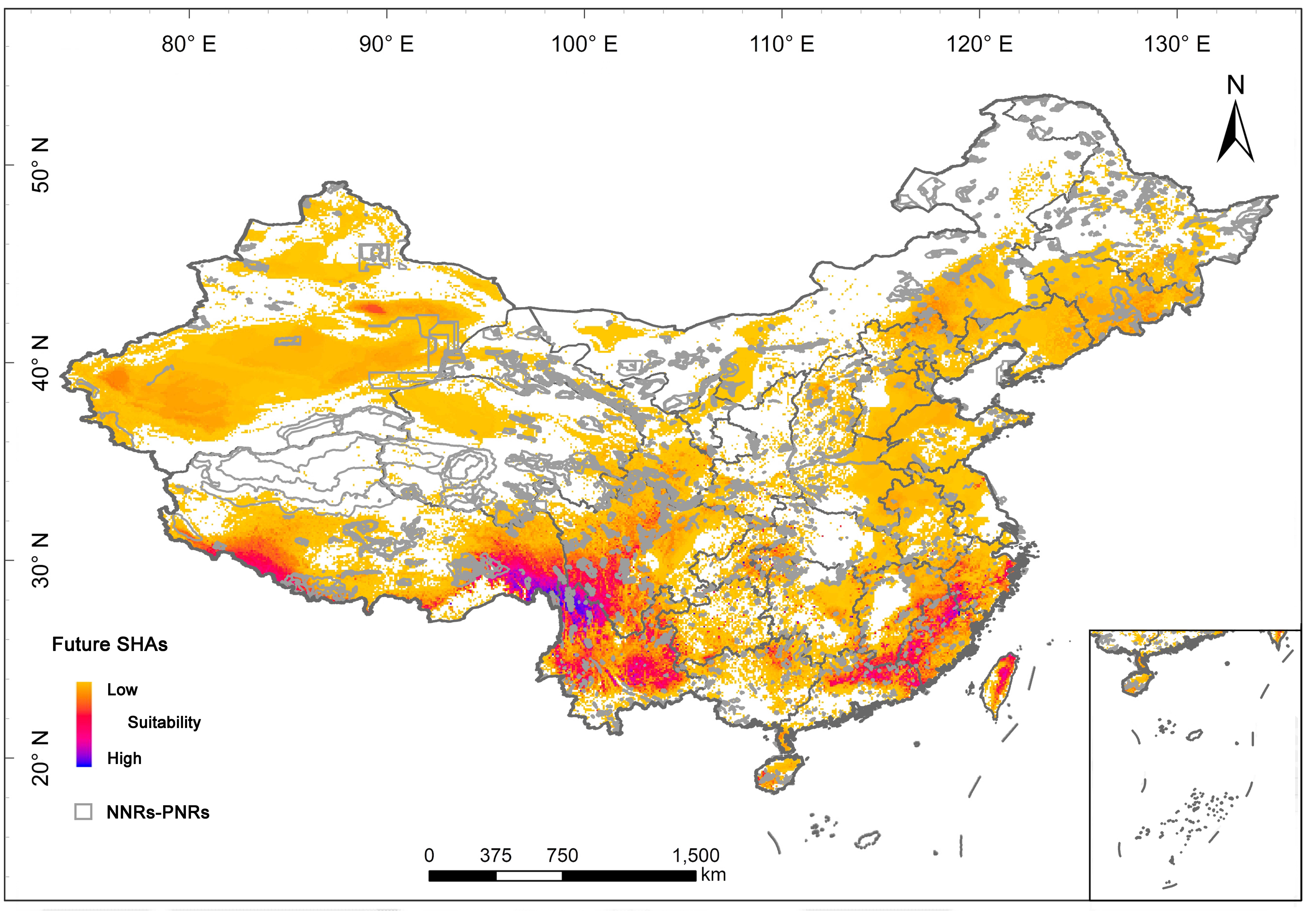

Supplement: Supplementary file 1 — Additional file 1: Fig. S1. Dragon Boat Festival wild medicinal plants market in Gongcheng, Guangxi China. Fig. S2. Map of the main mountains range in China. Fig. S3. Administrative division map of China (including the distribution of neighboring countries) (http://bzdt.ch.mnr.gov.cn/index.html). Fig. S4. The top 151 grids with highest species richness of all medicinal plants (AMPs_SR). Fig. S5. The top 152 grids with highest species richness of endemic medicinal plants (EMPs_SR). Fig. S6. The top 157 grids with highest species richness of threatened medicinal plants (TMPs_SR). Fig. S7. The top 156 grids with highest species richness of CITES listed medicinal plants (CMPs_SR). Fig. S8. The top 145 grids with highest species richness of national protected medicinal plants (NPMPs_SR). Fig. S9. The statistics of Areas under the Operating Characteristic Curve (AUC) of each threatened medicinal plants in MaxEnt. Fig. S10. Superposition of current and future suitable habitat areas with diversity hotspots and conservation gaps. Fig. S11. Superposition of current suitable habitat areas (current SHAs) with national and provincial nature reserves (NNRs-PNRs). Fig. S12. Superposition of future suitable habitat areas (future SHAs) with national and provincial nature reserves (NNRs-PNRs). [file 12915_2022_1285_MOESM1_ESM.doc]
