# Supplementary material for: Developing long-term conservation priority planning for medicinal plants in China by combining conservation status with diversity hotspot analyses and climate change prediction
Source: BMC Biol. 2022 Apr 21;20:89. doi: 10.1186/s12915-022-01285-4 (PMC9027417; doi:10.1186/s12915-022-01285-4)
Supplement: Supplementary file 2 — Additional file 2: Table S2.1. Hotspot areas of medicinal plants in China. Table S2.2. Species composition of different groups in China, diversity hotspots, conservation effectiveness and gaps for diversity hotspots, and grid cells covered by nature reserves focusing on the whole country. [file 12915_2022_1285_MOESM2_ESM.doc]

**Additional File 2. Supplementary Tables**

**(Tables S2.1 – S2.2)**

**Developing long-term conservation priority planning for medicinal plants in China by combining conservation status with diversity hotspot analyses and climate change prediction**

**Table S2.1.** Hotspot areas of medicinal plants in China. +++: major hotspot area; ++: medium hotspot area; +: minor hotspot area. The number of biodiversity hotspots refer to: ① Hengduan Mountains (western Sichuan), ② Bashan-Wushan Mountains, ③ Tianmu Mountains, ④ Hengduan Mountains (north-western Yunnan), ⑤ the junction area between Guizhou and Guangxi, ⑥ Nanling Mountains, ⑦ the Xishuangbanna region, ⑧ the boundary areas between Vietnam and China, ⑨ Hainan Island.

| Hotspots  Methods | ① | ② | ③ | ④ | ⑤ | ⑥ | ⑦ | ⑧ | ⑨ |
| --- | --- | --- | --- | --- | --- | --- | --- | --- | --- |
| Top 5% richness algorithm hotspots | +++ | +++ | ++ | +++ | +++ | +++ | ++ | +++ | ++ |
| Complementary algorithm hotspots | +++ | +++ | ++ | +++ | ++ | +++ | +++ | +++ | ++ |
| Diversity hotspots | +++ | +++ | ++ | +++ | +++ | +++ | +++ | +++ | ++ |

**Table S2.2.** Species composition of different groups in China, diversity hotspots, conservation effectiveness and gaps for diversity hotspots, and grid cells covered by nature reserves focusing on the whole country.

NRs = nature reserves, NNRs = national nature reserves and PNRs = provincial nature reserves

| **Taxa** | **China** | **Diversity hotspots** | **Conservation effectiveness for diversity hotspots** | | | **Conservation gaps for diversity hotspots** | | | **Grid cells covered by NRs focusing on the whole country** | | |
| --- | --- | --- | --- | --- | --- | --- | --- | --- | --- | --- | --- |
|  |  |  | **NNRs** | **PNRs** | **NNRs and PNRs** | **NNRs** | **PNRs** | **NNRs and PNRs** | **NNRs** | **PNRs** | **NNRs and PNRs** |
| **Threatened medicinal plants (TH)** | 620 | 580 | 536 | 508 | 560 | 423 | 503 | 321 | 589 | 570 | 606 |
| **Endemic medicinal plants excluded threatened medicinal plants (EN)** | 2910 | 2806 | 2716 | 2650 | 2775 | 2291 | 2552 | 1762 | 2829 | 2846 | 2888 |
| **Remaining species excluded threatened and endemic medicinal plants (REMA)** | 6226 | 5952 | 5638 | 5556 | 5800 | 5251 | 5624 | 4569 | 6013 | 6020 | 6147 |
| **Total number of medicinal plants** | 9756 | 9338 | 8890 | 8714 | 9135 | 7965 | 8679 | 6652 | 9431 | 9436 | 9641 |
